# A scorecard of progress towards measles elimination in 15 west African countries, 2001–19: a retrospective, multicountry analysis of national immunisation coverage and surveillance data

**DOI:** 10.1016/S2214-109X(20)30481-2

**Published:** 2021-02-16

**Authors:** Oghenebrume Wariri, Esin Nkereuwem, Ngozi A Erondu, Bassey Edem, Oluwatosin O Nkereuwem, Olubukola T Idoko, Emmanuel Agogo, Joseph E Enegela, Tom Sesay, Iya Saidou Conde, Landry Kaucley, Anthony Afum-Adjei Awuah, Sule Abdullahi, Richard Ray Luce, Richard Banda, Terna Nomhwange, Beate Kampmann

**Affiliations:** a Vaccines and Immunity Theme, MRC Unit the Gambia at the London School of Hygiene and Tropical Medicine, Fajara, The Gambia; b Centre for Universal Health, Chatham House, London, UK; c Sanofi Pasteur, Lyon, France; d The Vaccine Centre, London School of Hygiene and Tropical Medicine, London, UK; e Nigeria Centre for Disease Control, Abuja, Nigeria; f Africa Diseases Prevention and Research Development Initiative, Abuja, Nigeria; g Expanded Programme on Immunization, Freetown, Sierra Leone; h Expanded Programme on Immunization, Conakry, Guinea; i Expanded Programme on Immunization, Cotonou, Benin; j Kumasi Centre for Collaborative Research in Tropical Medicine, Kumasi, Ghana; k WHO Country Office, Monrovia, Liberia; l WHO, West African Regional Support Team, Ouagadougou, Burkina Faso; m WHO Country Office, Abuja, Nigeria

## Abstract

**Background:**

The WHO Regional Office for the Africa Regional Immunization Technical Advisory Group, in 2011, adopted the measles control and elimination goals for all countries of the African region to achieve in 2015 and 2020 respectively. Our aim was to track the current status of progress towards measles control and elimination milestones across 15 west African countries between 2001 and 2019.

**Methods:**

We did a retrospective multicountry series analysis of national immunisation coverage and case surveillance data from Jan 1, 2001, to Dec 31, 2019. Our analysis focused on the 15 west African countries that constitute the Economic Community of West African States. We tracked progress in the coverage of measles-containing vaccines (MCVs), measles supplementary immunisation activities, and measles incidence rates. We developed a country-level measles summary scorecard using eight indicators to track progress towards measles elimination as of the end of 2019. The summary indicators were tracked against measles control and elimination milestones.

**Findings:**

The weighted average regional first-dose MCV coverage in 2019 was 66% compared with 45% in 2001. 73% (11 of 15) of the west African countries had introduced second-dose MCV as of December, 2019. An estimated 4 588 040 children (aged 12–23 months) did not receive first-dose MCV in 2019, the majority (71%) of whom lived in Nigeria. Based on the scorecard, 12 (80%) countries are off-track to achieving measles elimination milestones; however, Cape Verde, The Gambia, and Ghana have made substantial progress.

**Interpretation:**

Measles will continue to be endemic in west Africa after 2020. The regional measles incidence rate in 2019 was 33 times the 2020 elimination target of less than 1 case per million population. However, some hope exists as countries can look at the efforts made by Cape Verde, The Gambia, and Ghana and learn from them.

**Funding:**

None.

## Introduction

Worldwide, more than 140 000 people died from measles in 2018, despite the availability of an effective, safe, and inexpensive measles vaccine licensed since 1963.^1^ The majority of these deaths occurred in sub-Saharan Africa.^1^ Measles is highly infectious and can infect up to 90% of susceptible contacts.^2^ Its high infectivity is underscored by its basic reproductive number (R_0_), which is an estimate of the number of people in a susceptible population that can acquire the disease from a single source. Currently, the world is rightly immersed in combating infectious disease emergencies such as COVID-19 (estimated R_0_ of 1–3)^3^ and Ebola virus disease (estimated R_0_ of 2).^4^ Measles virus infection, with an exponentially higher R_0_ of 12–18, is completely preventable with two doses of measles-containing vaccine (MCV), but can be spread faster if populations are not sufficiently vaccinated.^5^ Although measles virus infection could be self-limiting, in some instances, debilitating post-disease sequelae including blindness, deafness, and impairment of the immune system occur for months afterwards, which leaves children susceptible to other deadly infectious diseases.^6^

In 2012, the American Red Cross, US Centers for Disease Control and Prevention, UNICEF, UN Foundation, and WHO targeted measles for elimination in five of six WHO regions by 2020.^7^ The key strategies were to ensure high vaccination coverage with routine vaccinations (ie, MCV first dose [MCV1] and second dose [MCV2]), campaign-based supplementary immunisation activities (SIAs) to target subpopulations to rapidly scale up coverage,^8^ and active case-based surveillance.^7^ Consequently, there was a 66% reduction in annual measles incidence, a 73% reduction in annual measles mortality, and 23·3 million measles deaths were prevented between 2000 and 2018.^9^ Despite this progress, the elimination targets are far from being met due to vaccine demand-related issues prevalent in high-income countries, recurrent conflicts, and weak immunisation systems in low-income countries.^9^

**Research in context**

**Evidence before this study**

We searched for studies investigating trends in measles elimination or control in west Africa in the past 2 decades, from Jan 1, 2001, to Dec 31, 2019, using PubMed. We used the search terms “measles” [all fields] AND “West Africa” [all fields] OR “ECOWAS” [all fields] AND “elimination OR control” [all fields]. No language restrictions were applied. We included studies that were based in west Africa or included at least one west African country. Most studies were active case-based surveillances or descriptive analyses of routinely collected data. The studies focused on either one region within a country, an entire country, or comparisons between two and 15 countries. These studies investigated ongoing measles outbreaks, assessed routine measles vaccine coverages, and assessed the coverage and effectiveness of supplementary immunisation activities (SIAs) using routinely collected, publicly available data sources. We were, however, unable to assess the quality of the data used in the active case-based surveillance studies.Previous studies reported increasing trends in measles cases in the region following the 2014–16 Ebola epidemic in the region. Studies also projected an accumulation of a large cluster of children unvaccinated for measles across Guinea, Liberia, and Sierra Leone and an expected increase in the size of regional outbreaks projected to result in 2000–16 000 additional deaths. A study in Nigeria reported that the national coverage of the first dose of measles-containing vaccine (MCV1) was consistently low between 2012 and 2015, with wide variations between regions within the country. A study also reported that despite their high routine MCV1 coverage, most west African countries have some programme gaps indicating that they do not meet all the criteria to undergo verification of elimination at this point. In a report of MCV1 coverage estimates in 2015, only two countries in the region had coverage of 95% or more for both MCV1 and the second dose of measles-containing vaccine (MCV2) whereas five countries had coverage of more than 80% for both doses. Dropout rates of more than 20% between MCV1 and MCV2 existed in 12 west African countries. The report across most studies showed that the MCV2 coverage remains low in the region.The evidence also showed that, in some countries, measles SIAs appeared to have successfully reached a higher proportion of measles vaccine-naive children compared to others. Across all countries, SIAs played an important part in reaching children from poor households, reducing the overall magnitude of the measles virus outbreak in 2009. A study from Côte d’Ivoire and Burkina Faso found that migration of children between both countries played a major role in the failure of SIAs to interrupt measles transmission.

**Added value of this study**

This is the first comprehensive analysis, to our knowledge, of progress towards the global measles control and elimination milestones between 2001 and 2019, exclusively focused on 15 west African countries. The weighted mean MCV1 coverage across the region increased from 45% in 2001 to 66% in 2019. However, Benin, Côte d’Ivoire, Guinea, and Guinea Bissau were yet to introduce the recommended MCV2 vaccination as of December, 2019. There were an estimated 4 588 040 children (aged 12–23 months) in the region who did not receive MCV1 in 2019; the majority (71%) lived in Nigeria. In this analysis, we propose a scorecard that highlights key metrics which could be used to categorise, track, and compare country-level progress towards measles elimination. Based on this, 12 (80%) countries in the region are off-track to achieving measles elimination milestones, while Cape Verde, The Gambia, and Ghana have made substantial progress.

**Implications of all the available evidence**

The available evidence shows that there has been substantial progress in MCV1 coverage, MCV2 introduction, and the reach of SIAs across the west African region between 2001 and 2019. Although there has been an overall reduction in the incidence of measles cases per million population, there still exist inequalities within and between countries in the region. The region did not meet the measles control milestones of 2015 and is currently off-track to achieve the 2020 elimination milestones. Although Cape Verde, The Gambia, and Ghana have made substantial strides, the results suggest that, as things stand, measles will continue to be endemic in the region well into the future in a landscape of a visa-free cross-border migration. These findings should inform regional strategies and shared learning, which could be coordinated by the region for countries to strengthen their routine immunisation systems, and ensure local ownership and continued financial commitment. Furthermore, countries must conduct well targeted and integrated SIAs to reduce the inequality gaps, improve case-based surveillance to ensure rapid detection, and enhance community engagement to address any vaccine demand-related issues. These actions remain even more important as more countries within the region graduate from eligibility for Gavi, the Vaccine Alliance, support.

In 2011, the WHO Regional Office for Africa Regional Immunisation Technical Advisory Group adopted the *Measles elimination: a strategy for the African Region*^10^ for all member states of the African region of the WHO (AFRO) by 2020. As part of this strategy, a measles control milestone was set for WHO AFRO countries by 2015. The milestones for measles control were to reduce the annual measles incidence rate to less than five cases per million, increase MCV1 and MCV2 coverage to 90% or more nationally, and 80% or more in all districts, and to achieve 95% or more coverage during measles SIAs.^7^ Furthermore, measles elimination milestones were set for the end of 2020. These were, specifically, to achieve and maintain annual measles incidence of less than one case per million population nationally, 95% or more MCV1 and MCV2 coverage nationally and in all districts, and at least 95% coverage in all scheduled or outbreak-related measles SIAs, among other targets.^10^

Despite these ambitious elimination targets, since 2019, the world's largest single-nation measles outbreak has persisted in Democratic Republic of the Congo, with more than 310 000 suspected cases, and resulted in the death of more than 6000 people as of January, 2020.^11^ In west Africa, the 2014–16 Ebola outbreak in Guinea, Liberia, and Sierra Leone had a crippling effect on immunisation systems, leaving behind an additional public health crisis of a large connected cluster of measles vaccine-naive children.^12^ The regional risk of sustained measles transmission is thought to have been further compounded by recurrent humanitarian crises, which have caused displacement of people across transnational borders.^13^ In this landscape, we set out to assess the region's trajectory a decade before the measles elimination and control strategy was adopted. We then followed up the progress for a subsequent decade after the adoption of the elimination and control strategy to determine if the measles control milestones were achieved in 2015 and to track the current trajectory towards the measles elimination milestones in west Africa.

## Methods

### Study design

We did a retrospective multicountry series analysis of national measles immunisation coverage and case surveillance data from Jan 1, 2001, to Dec 31, 2019. This analysis focused on the 15 west African countries that constitute the Economic Community of West African States (ECOWAS). The ECOWAS is an alliance whose aim is to promote regional integration and allow visa-free trans-border migration for all west Africans.^14^ The 15-member regional bloc comprises Benin, Burkina Faso, Cape Verde, Côte d’Ivoire, The Gambia, Ghana, Guinea, Guinea Bissau, Liberia, Mali, Niger, Nigeria, Senegal, Sierra Leone, and Togo. In 2019, the region had an estimated population of 387 million, with Nigeria accounting for about 52%.^15^ In 2019, Benin, Cape Verde, Côte d’Ivoire, Ghana, Nigeria, and Senegal were lower-middle-income countries, whereas the remaining nine countries were low-income countries.^16^ All ECOWAS countries apart from Cape Verde receive Gavi, the Vaccine Alliance support, for their immunisation system, with some of these countries scheduled for transition from Gavi support in the coming years.^17^

### Data sources

We obtained data from the UN World Population Prospects 2019,^15^ WHO/UNICEF Estimates of National Immunization Coverage (WUENIC) database,^18^ Multiple Indicator Cluster Survey (MICS),^19^ and Demographic and Health Survey (DHS; table).^20^ We used the final official yearly reports of the WHO measles surveillance database from 2001 to 2019,^21^ and periodic measles SIAs from WHO's database.^22^ We also extracted the Joint External Evaluation (JEE) scores for each country.^23^ The JEE score uses measles vaccine coverage as one of its measures to assess country-level capacity to prevent, detect, and rapidly respond to public health risks.^23^

**Table:** Summary of the data used in this analysis, their respective sources, and years extracted per country

	**Data extracte**d	**Years extracted**	**Date accessed**
UN World Population Prospects 2019^15^	Country-level estimates of total population and surviving infants for all 15 ECOWAS countries	2001–19	July 15, 2020
WHO/UNICEF Estimates of National Immunization Coverage database^18^	Country-level MCV1 and MCV2 coverage estimates for all 15 ECOWAS countries	2001–19	July 15, 2020
WHO measles surveillance database^21^	Absolute number of measles cases reported per country for all 15 ECOWAS countries	2001–19	July 15,2020
MICS^19^*	Country-level MCV1 coverage disaggregated by geographical areas	2014–18	July 18, 2020
DHS^20^*	Country-level MCV1 coverage disaggregated by geographical areas	2005–18	July 18, 2020
WHO Immunization Database^22^	Country-level supplementary immunisation activities for all 15 ECOWAS countries	2001–19	July 21, 2020
JEE mission reports^23^†	JEE scores per country	2016–19	July 21, 2020

DHS=Demographic and Health Survey. ECOWAS=Economic Community of West African States. JEE=Joint External Evaluation. MCV1=first dose of measles-containing vaccine. MCV2=second dose of measles-containing-vaccine. MICS=Multiple Indicator Cluster Survey.

* Data from either the MICS or DHS were extracted depending on which survey was the most recent in the country: Benin (DHS 2017–18), Burkina Faso (DHS 2010), Cabo Verde (DHS 2005), Côte d'Ivoire (MICS 2016), The Gambia (MICS 2018), Ghana (DHS 2014), Guinea (DHS 2018), Guinea Bissau (MICS 2014), Liberia (DHS 2013), Mali (DHS 2018), Niger (DHS 2012), Nigeria (DHS 2018), Senegal (DHS 2017), Sierra Leone (MICS 2017), and Togo (DHS 2013–14).

† 2016=Côte d'Ivoire, Liberia, Senegal, and Sierra Leone. 2017=Benin, Burkina Faso, The Gambia, Ghana, Guinea, Mali, and Nigeria. 2018=Niger and Togo. 2019=Guinea Bissau.

### Estimating MCV1 coverage trends, population reached by SIAs, and MCV2 introductions

We obtained MCV1 coverage data from the WUENIC database.^18^ We computed the regional median MCV1 coverage and mean MCV1 coverage weighted by each country's corresponding annual surviving infant population from 2001 to 2019 and plotted a line graph showing the trends. Furthermore, the highest and lowest MCV1 coverage countries for the corresponding year were illustrated by creating two trend lines representing the countries.

To estimate the reach of SIAs, we compared the absolute number of the population targeted by SIAs across the 15 west Africa countries against the actual population reached using SIA data from the WHO's database.^22^ We tracked the number of countries that introduced MCV2 per year across west Africa between 2001 and 2019 using data from the WUENIC database.^18^

### Estimating the number of children who were unvaccinated for MCV1 in 2019

We estimated the absolute number of children unvaccinated for MCV1 as the proportion of the 2019 country-specific surviving infants (based on the UN World Population Prospects 2019)^15^ who did not receive MCV1 (based on the WUENIC database).^18^ We illustrated this using a bubble map, with the size of each bubble representing the number of children unvaccinated with MCV1.

### Estimating country-level equity in MCV1 vaccination coverage

We obtained the most recently available disaggregated MCV1 coverage from the MICS and DHS data.^19^, ^20^ This was subsequently mapped according to country-level subnational administrative areas into MCV1 coverage categories of less than 20%, 20–39%, 40–59%, 60–79%, 80–94%, and 95% or more. The WHO considers equity as an important component of progress towards elimination of measles.^24^ Therefore, we estimated the most recent situation of the intracountry geographical equity gap in MCV1 coverage using a single summary measure of the difference as a proxy for absolute inequity by subtracting MCV1 coverage in the subnational administrative area with the lowest from that with the highest per country (in percentage points). We considered an absolute coverage gap of 20% or less as the reference equity benchmark.^24^

### Estimating the number of measles cases

The measles cases were the absolute number of laboratory-confirmed, epidemiologically confirmed, and clinically confirmed cases reported to WHO per country.^25^ WHO defines laboratory-confirmed measles cases as cases that meet the clinical case definition with the presence of measles-specific IgM antibodies, and epidemiologically confirmed measles cases as cases that meet the clinical case definition and are linked to a laboratory-confirmed case. Clinically confirmed measles cases are defined as cases that meet the clinical case definition and for which no adequate blood specimen was taken. A clinical case definition included any person in whom a clinician suspects measles infection, or any person with fever and maculopapular rash (ie, non-vesicular) and cough, coryza (ie, runny nose), or conjunctivitis (ie, red eyes).^25^

### Estimating the annual measles incidence rate in west Africa

Annual measles incidence rate per million was calculated by dividing the number of measles cases per year by corresponding national population estimates. The regional measles incidence rate was, therefore, the absolute number of yearly measles cases in west Africa divided by the total national population of the 15 countries in that same year. We illustrated the highest and lowest measles incidence rates in the region for the corresponding year by creating two trend lines representing the country with the highest and lowest incidence rates.^10^

### Comparing MCV1 coverage, SIAs, and measles incidence rate

We plotted a trend line of the mean regional MCV1 coverage (weighted by the total regional surviving infant population per country) and measles SIAs against the mean regional measles incidence rate (weighted by the total population per country). Through this approach, we highlighted the contribution of increasing and decreasing MCV1 coverage and SIA activities on measles incidence rates.

### Assessing progress towards measles elimination (country-level measles summary scorecard)

To assess each country's progress towards measles elimination, we developed a country-level measles summary scorecard as of the end of 2019. The scorecard was developed in response to the 2016 Mid-term Review of the Global Measles and Rubella Strategic plan 2012–2020 that encouraged countries to develop standard methods to categorise progress and the likelihood of achieving the measles milestones.^26^ The scorecard comprised eight country-level indicators: 2015 MCV1 coverage (tracked against the 90% control milestone); MCV1 coverage as of 2019 (tracked against the 95% elimination milestone); 2015 MCV2 coverage (tracked against the 90% control milestone); MCV2 coverage as of 2019 (tracked against the 95% elimination milestone); geographical equity gap in MCV1 coverage (tracked against the benchmark of ≤20% points);^24^ 2015 measles incidence rate (tracked against the control milestone of <5 cases per million); measles incidence rate as of 2019 (tracked against the elimination milestone of <1 case per million);^10^ and JEE scores, reported as dark green for scores 4 and 5, yellow for scores 2 and 3, and red for score 1.^23^

According to WHO and UNICEF, MCV1 and MCV2 coverage was categorised as dark green if at least 90% in 2015, or at least 95% in 2019; light green if 80–89% in 2015 and 80–94% in 2019; yellow if 50–79%; or red if 50% or less. Countries yet to commence MCV2 as of December, 2019, were also categorised as red. Measles incidence rate per million was categorised as dark green if for less than 5 cases per million in 2015 and less than 1 case per million in 2019; light green if 5–9 cases per million in 2015 and 1–4 cases per million in 2019; yellow if 10–19 cases per million in 2015 and 5–9 cases per million in 2019; and red if more than 20 cases per million in 2015 and more than 10 cases per million in 2019. Intracountry geographical equity gap was categorised as dark green if 20% or less points and red if more than 20% points. JEE scores were categorised as red if 1, yellow if 2–3, and dark green if 4–5.

All eight indicators were weighted equally, giving a total score of eight. We considered a country that achieved dark or light green in at least six indicators to have made substantial progress. Countries that achieved dark or light green in three to five indicators were considered to have made borderline progress. In contrast, countries that achieved two or less were considered to have made little progress towards measles elimination.

We did a mini validation of the scorecard using data from countries in the WHO South-East Asia region. We selected this region because the measles virus infection is endemic, and MCV1 and MCV2 are routinely administered. All countries with complete dataset required to generate the scorecard were included. We calculated the sensitivity and specificity of the scorecard for correctly identifying countries with substantial progress, when compared with the WHO measles elimination certification for those countries as gold standard. The scorecard showed a 75% sensitivity and specificity (appendix pp 2–17).

All figures were generated using Microsoft Excel (Microsoft, Seattle, WA, USA), whereas the disaggregated MCV1 coverage map was created using ArcGIS software (version 10.4, Environmental Systems Research Institute Redlands, CA, USA).

### Role of the funding source

The funders of the study did not have any role in study design, data collection, data analysis, data interpretation, or writing of the manuscript.

## Results

**Figure 1:**
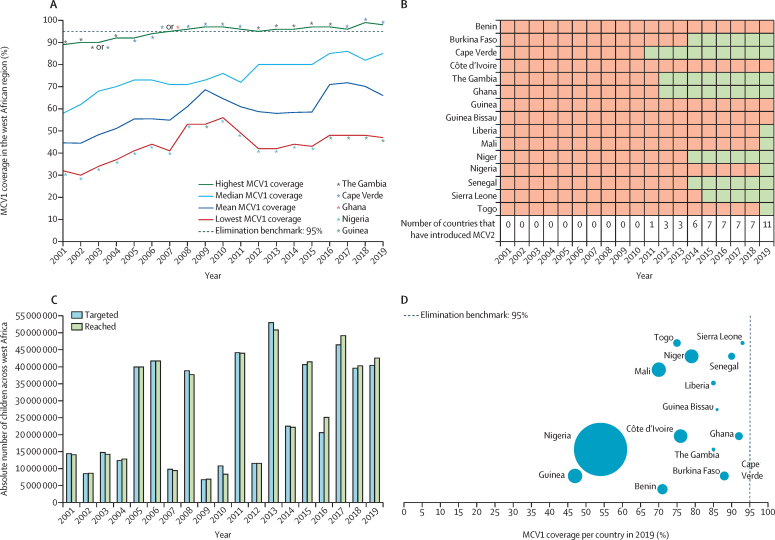
MCV1 coverage, MCV2 introduction, reach of supplementary immunisation activities, and unvaccinated children in west Africa, 2001–19 (A) Regional median and weighted mean MCV1 coverage in west Africa between 2001 and 2019. Mean MCV1 coverage weighted by each country's total surviving infant population as a proportion of the surviving infant population in west Africa per year. (B) MCV2 introduction in west Africa, 2001–19. (C) Cumulative population targeted and reached by supplementary immunisation activities in west Africa, 2001–19. (D) Number of west African children who did not receive MCV1 vaccination per country in west Africa in 2019; the size of each bubble corresponds to the absolute number of unvaccinated children per country in 2019. MCV1=first dose of measles-containing vaccine. MCV2=second dose of measles-containing vaccine.

Overall, MCV1 coverage increased across west Africa between 2001 and 2019. The weighted mean MCV1 coverage was 45% in 2001 and increased to an initial peak in 2009 (69%). However, this increase in weighted mean MCV1 coverage was followed by an average yearly decline of 2% until it reached a coverage of 59% in 2015 before rising sharply to 72% in 2017 and declined afterwards (figure 1A). The highest country-level MCV1 coverage ranged from 89% in 2001 to 99% in 2019, with Cape Verde and The Gambia consistently reporting the highest coverage. The first west African country to introduce MCV2 was Cape Verde in 2011, followed by The Gambia and Ghana in 2012. By 2019, 73% (11 of 15) of countries in the region had introduced MCV2; Benin, Côte d’Ivoire, Guinea, and Guinea-Bissau had not yet introduced the vaccine (figure 1B). The population targeted for SIAs in the region increased consistently between 2009 and 2019 with the actual population reached surpassing the targeted population during the same period (figure 1C). There were an estimated 4 588 040 children (aged 12–23 months) across the 15 west African countries who did not receive MCV1 in 2019. A majority (71%) of these children lived in Nigeria, which also accounted for 53% of the regional 13 474 000 surviving infant population in 2019 (figure 1D).

**Figure 2:**
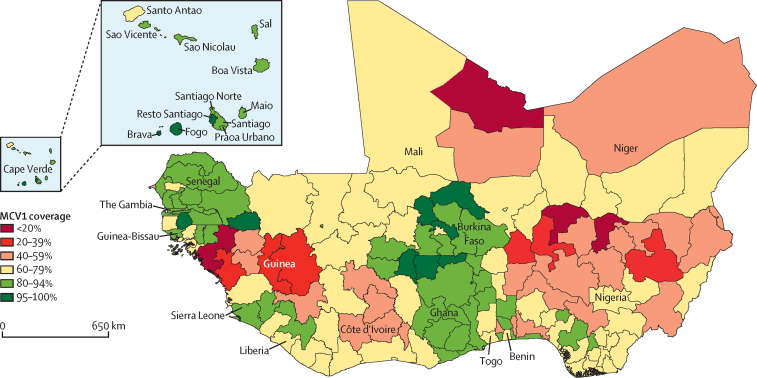
Map of west Africa showing disaggregated MCV1 coverage (%) by subnational administrative areas (regions or states) across the 15 ECOWAS countries The most recently available, disaggregated national survey data used per country. Benin (DHS 2017–18), Burkina Faso (DHS 2010), Cape Verde (DHS 2005), Côte d’Ivoire (MICS 2016), Gambia (MICS 2018), Ghana (DHS 2014), Guinea (DHS 2018), Guinea Bissau (MICS 2014), Liberia (DHS 2013), Mali (DHS 2018), Niger (DHS 2012), Nigeria (DHS 2018), Senegal (DHS 2017), Sierra Leone (MICS 2017), and Togo (DHS 2013–14). DHS=Demographic and Health Survey. ECOWAS=Economic Community of West African States. MCV1=first dose of measles-containing vaccine. MICS=Multiple Indicator Cluster Survey.

**Figure 3:**
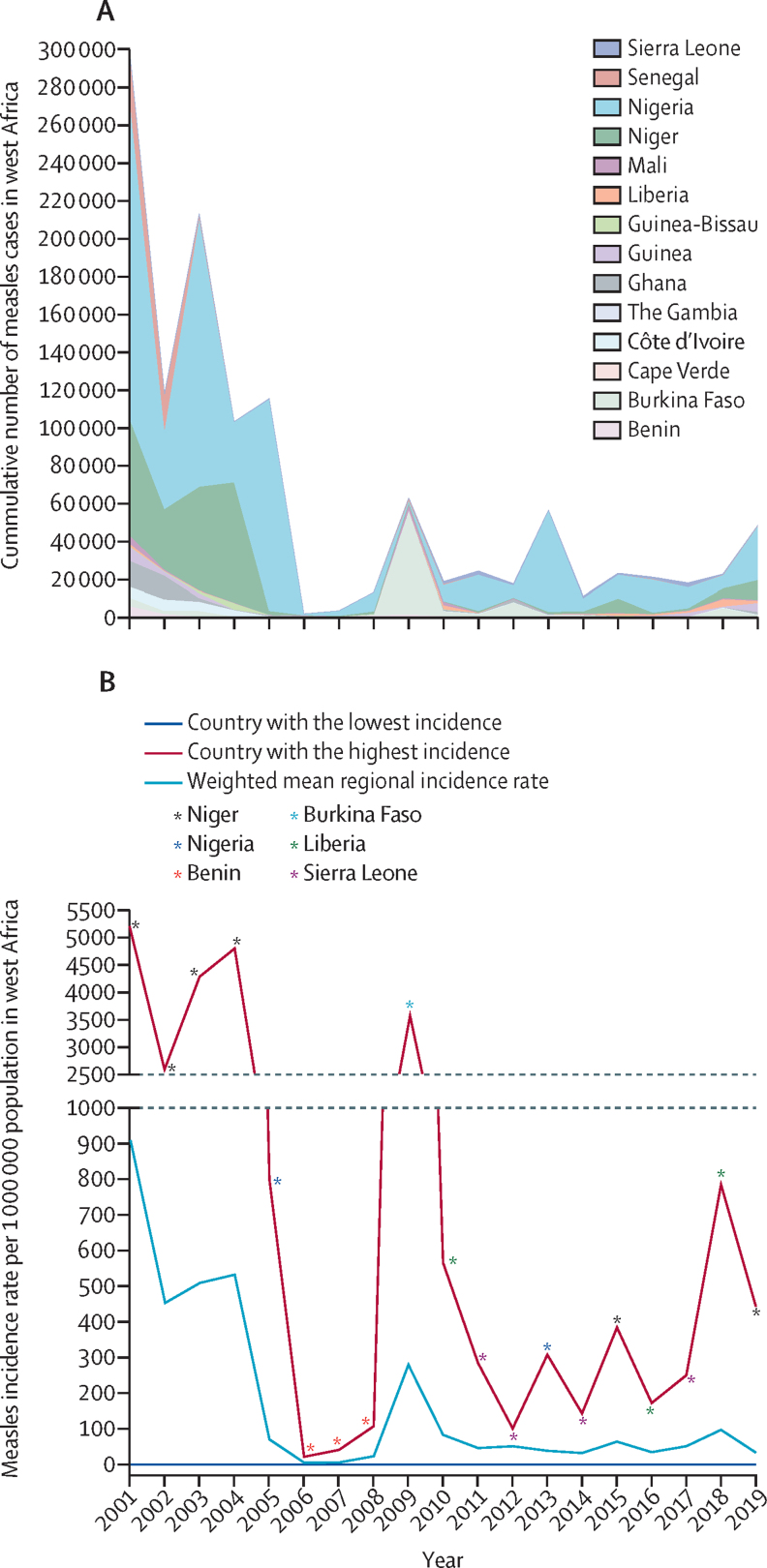
Measles cases and measles incidence rate per million population in west Africa, 2001–19 (A) Cumulative number of reported measles cases per year. The number of measles cases for Togo during this period were quite small compared with other countries, thus, they are not visible in this figure. (B) Measles incidence rate per million population between 2001 and 2019 based on available country-level cases reported to WHO in 15 west African countries. Cape Verde consistently reported the lowest measles incidence rate of zero per million population. Country-level population was based on the UN World Population Prospects 2019.

Of the total 166 subnational administrative areas across the 15 west African countries, 11 (7%) had achieved MCV1 coverage of at least 95%. The 11 subnational administrative areas were Resto Santiago, Fogo, and Brava in Cape Verde; Nord, Plateau Central, Sahel, and Sud-Ouest regions in Burkina Faso; Upper East and Upper West regions in Ghana; and Kedougou and Sedhiou regions in Senegal. More than 80% of the subnational administrative areas in Cabo Verde (11 of 12 regions), Burkina Faso (11 of 13 regions), The Gambia (all regions), Ghana (nine of ten regions), and Senegal (11 of 14 regions) had MCV1 coverage of at least 80% (figure 2). The highest cumulative number of measles cases was 299 361 reported in 2001, whereas the lowest was 1709 measles cases reported in 2006 (figure 3A). The number of cases almost tripled between 2017 and 2019 (17 653 in 2017 *vs* 47 819 cases in 2019). There were no measles cases reported in Cape Verde between 2001 and 2019. The weighted mean measles incidence rate ranged between 909 cases per million to six cases per million population in west Africa (figure 3B). The lowest (six cases per million) weighted mean incidence rate was in 2006 and 2007, whereas the highest (909 cases per million) was in 2001. Benin, Liberia, Niger, and Sierra Leone repeatedly had the highest measles incidence rate in the period under study.

**Figure 4:**
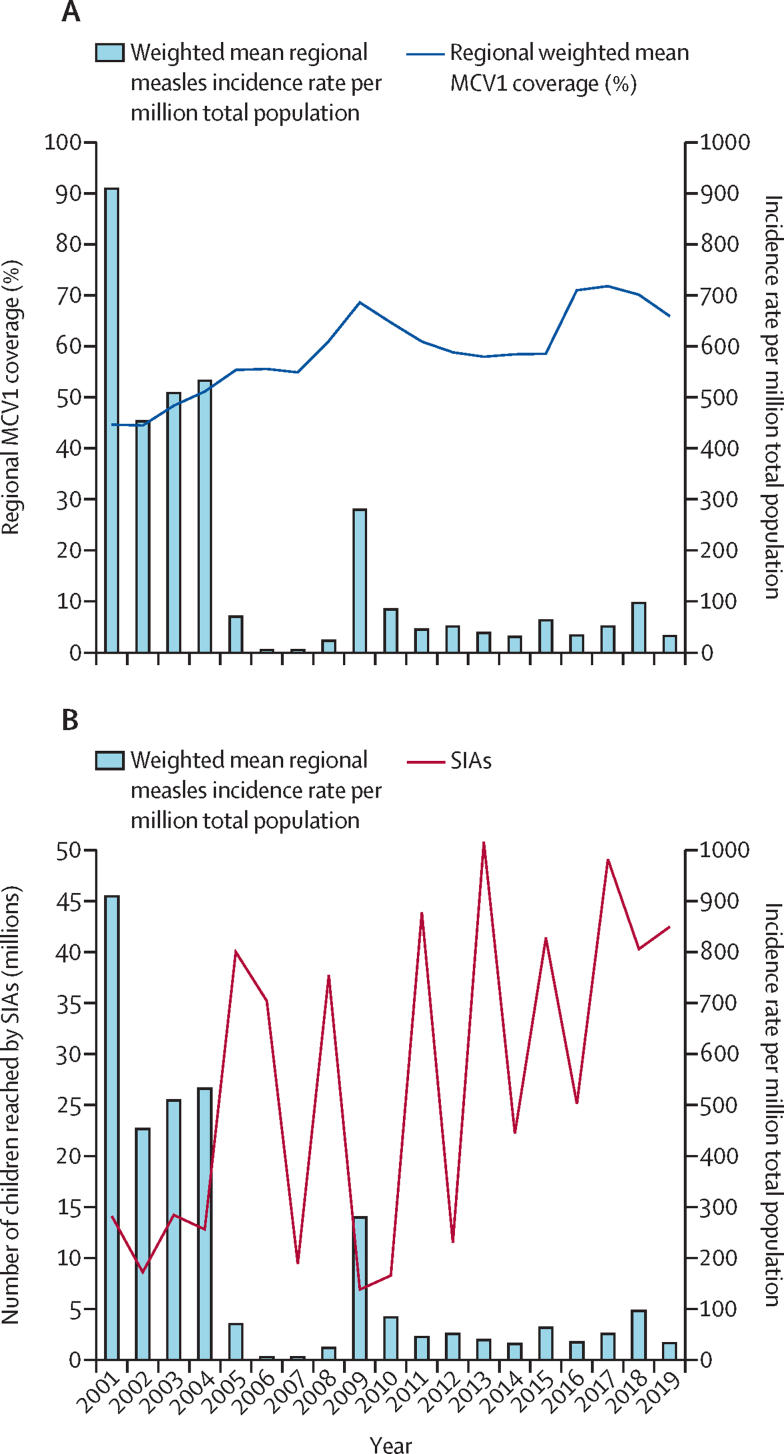
Relationship between MCV1 coverage, SIAs, and measles incidence rate per million population in west Africa, 2001–19 (A) Relationship between weighted mean MCV1 coverage and weighted mean regional measles incidence rate in west Africa, 2001–19. (B) Relationship between measles SIAs and weighted mean measles incidence rate in west Africa, 2001–19. Some countries did more than one SIA per year. MCV1=first dose of measles-containing vaccine. SIAs=supplementary immunisation activities.

Overall, there was an inverse relationship between the weighted mean MCV1 coverage and weighted mean measles incidence rate (figure 4A). As the weighted mean MCV1 coverage increased between 2001 and 2019, the weighted mean measles incidence rate per million decreased. Similarly, an inverse relationship was also observed between the number of children reached by SIAs and the weighted mean measles incidence rate in west Africa (figure 4B).

**Figure 5:**
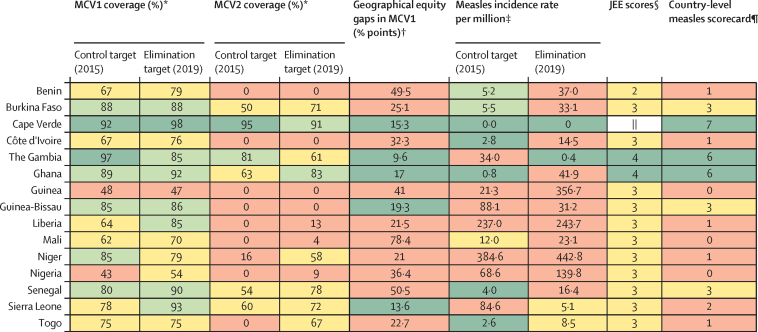
Scorecard summary showing achievement of measles elimination milestones across countries in the west African region JEE=Joint External Evaluation. MCV1=first dose of measles-containing vaccine. MCV2=second dose of measles-containing-vaccine. *MCV1 and MCV2 coverage categories: dark green=≥90% in 2015 and ≥95% in 2019; light green=80–89% in 2015 and 80–94% in 2019; yellow=50–79%; and red=≤50%. As of December, 2019, Benin, Côte d’Ivoire, Guinea, and Guinea-Bissau had not yet commenced MCV2 immunisation. †Intracountry geographical equity gap categories: dark green=≤20% points and red=>20% points. ‡Measles incidence rate per million categories: dark green=<5 cases per million in 2015 and <1 case per million in 2019; light green=5–9 cases per million in 2015 and 1–4 cases per million in 2019; yellow=10–19 cases per million in 2015 and 5–9 cases per million in 2019; and red=>20 cases per million in 2015 and >10 cases per million in 2019. §JEE categories: red=1; yellow=2–3; and dark green=4–5. ¶Country-level measles scorecard categories: dark green=achieved 6–8 of the 8 measles summary indicators; yellow=achieved 3–5 of the 8 measles summary indicators; and red=achieved only 1 or 2 of the 8 measles summary indicators. ||JEE yet to be done.

Cape Verde, The Gambia, and Ghana made substantial progress towards measles elimination (figure 5). On the other hand, Benin, Burkina Faso, Côte d’Ivoire, Guinea, Liberia, Mali, Niger, Nigeria, and Sierra Leone have made little progress towards measles elimination having achieved the benchmark of only one or two of the eight measles summary indicators.

## Discussion

To our knowledge, this analysis represents the first comprehensive regional level estimation of progress towards measles elimination targeted at the 15 ECOWAS countries. Since 2001, the region has made progress in increasing MCV1 uptake, introducing MCV2 across many countries, increasing the reach of measles SIAs, and reducing the incidence rate of measles per million population. Nevertheless, progress has been far from universal, even though most of the countries in the region have received Gavi support since 2000 to finance strategies to address country-level immunisation gaps. Measles control milestones were not met in most countries in 2015 and progress towards the elimination milestones are off-track in the region. Substantial variation within and between countries persist in MCV1 coverage, MCV2 introduction, and measles incidence rates, with, for example, regional measles incidence rate as of 2019 being more than 33 times the elimination target of less than 1 per million population by the end of 2020.

Cape Verde, The Gambia, and Ghana have made substantial progress and are on track to achieving the measles elimination milestones by 2020. This finding aligns with previous research on immunisation system performance in the region, which reported that Cape Verde and The Gambia had achieved the Global Vaccine Action Plan immunisation coverage and dropout targets since 2015.^27^ Other factors might explain the progress made by Cape Verde, The Gambia, and Ghana. Cape Verde and The Gambia are less populated (0·54 million people in Cape Verde and 2·1 million people in The Gambia)^15^ than other countries in the region. The majority of Gambians live within a convenient distance from a health facility, and the government regularly provide immunisation services using outreach clinics to target hard-to-reach subpopulations.^28^ This unique characteristic makes the delivery of immunisation services more efficient. This is seen in southeast Asia where Bhutan (population 0·76 million people), Maldives (0·54 million), and Timor-Leste (1·3 million),^15^ are the three of five countries that have eliminated measles in that region.

The population of a country, however, only provides a partial explanation for progress towards measles elimination. Ghana has a comparable population size to Côte d’Ivoire, which has made limited progress towards achieving the elimination milestones.^15^ Compared to other countries in the region, the health system in Ghana is considered well integrated and well financed, and implements up-to-date policies aimed at providing essential services, including immunisation.^29^ For example, Ghana is currently implementing the third comprehensive multi-year plan to provide strategic direction for immunisations in the country to meet national priorities and global targets. The current (2015–19) comprehensive multi-year plan being implemented for the Expanded Programme on Immunization follows the successful implementation of previous plans which have improved access and use of immunisation services in the country.^30^

Benin, Burkina Faso, Côte d’Ivoire, Guinea, Liberia, Mali, Niger, Nigeria, and Sierra Leone have made little progress towards achieving the global measles elimination milestones. In most of these countries, a complex mix of suboptimal MCV1 and MCV2 coverage, non-introduction of MCV2, a wide intracountry geographical equity gap in MCV1 coverage, and a double-digit measles incidence rate per million population persist. In 2001, WHO designated these nine countries among others as priority countries for measles elimination.^7^ Nonetheless, the pervasive measles-related issues seem to have persisted after almost two decades of adopting the ambitious strategic plan. With an overwhelming proportion of the estimated 4·5 million measles vaccine-naive children in west Africa resident in these nine countries, the region faces an ongoing, potentially widespread, and colliding epidemic of measles outbreaks.

Ongoing humanitarian crises pose one of the biggest obstacles to achieving the measles elimination targets in Benin, Burkina Faso, Côte d’Ivoire, Guinea, Liberia, Mali, Niger, Nigeria, and Sierra Leone.^26^ UNICEF estimate that two-thirds of the world's unvaccinated children live in conflict-affected countries.^31^ Burkina Faso, Mali, and Niger have experienced repeated deadly armed conflicts over the past decade. For more than a decade, Nigeria has struggled to curtail violent conflict orchestrated by the terrorist group Boko Haram. The ensuing humanitarian crises adversely impacted the measles elimination strategies due to massive population movement within and across national borders, and disrupted the delivery of immunisation services.^32^ Guinea, Liberia, and Sierra Leone, the three countries at the epicentre of the 2014–16 Ebola outbreak, were already suffering from low vaccination coverage rates. In the wake of the outbreak, several factors led to the disruption in routine immunisation services and underperformance of measles vaccination campaigns leaving behind a large cohort of unvaccinated children. For example, in Guinea, routine immunisation was suspended in many places with active Ebola virus transmission. Also, SIA campaigns were less effective in some communities that struggled with mistrust of public health professionals or reduced health seeking behaviour due to the fear of being infected with the virus at the health facility—a phenomenon that we are seeing during the ongoing COVID-19 pandemic.^33^

Our findings reflect the broader reality in sub-Saharan Africa. The 2016 mid-term review of the Global Measles and Rubella Strategic Plan 2012–2020 showed that despite some impact in AFRO, substantial challenges in reaching measles elimination remain.^26^ Among these challenges was recognition of the epidemiological shift in disease towards older age groups (adolescents and adults) that requires a parallel programmatic response.^34^ The 2015 revised African regional guidelines use the WHO-wide guidelines and does not address this shift within routine immunisation schedules.^35^ A beyond-2020 strategy should address this issue, must prioritise closing immunisation gaps, and focus on timely vaccination of all susceptible age groups, especially in areas of long-lasting low coverage.

The realities of concurrent outbreaks, humanitarian crises, and political unrest will continue to affect measles coverage. Future plans must not only acknowledge these challenges but reinforce strategies within special circumstances accordingly. Like polio, to actualise measles elimination, investments must be coordinated, consistent, and steady from both national governments as well as international partners.^36^ Compellingly, a clear challenge to this region is the poor uptake of MCV1 in Nigeria. With its high proportion of infants, this failure highly skews the measles coverage performance in west Africa. A combination of overall low national immunisation coverage and a geographical imbalance of four in every five measles cases being from the northern region, requires targeted and innovative approaches to increase coverage rates.^37^ Other approaches to improving coverage in Nigeria and similarly high-burden countries include spatial analysis of coverage gaps to inform more targeted campaigns as well as a successful transition of polio infrastructure to shore-up measles surveillance as vertical polio programmes diminish following the recent wild polio-free certification in the African region.

The datasets and methods used in this analysis might be subject to some limitations. First, the DHS and MICS immunisation coverage data used to generate the disaggregated MCV1 coverage map were survey-based, collected from vaccine cards and maternal recall. Although the DHS and MICS surveys provide disaggregated immunisation coverage data at subnational and district level compared with the non-disaggregated WUENIC coverage estimates, they are also prone to recall bias. Second, the DHS and MICS data used were from varying years (2005–18). Although the varying years of the data might limit direct temporal comparison in terms of the current status of in-country equity in MCV1 coverage, we only resorted to the best available data sources. Third, there are gaps in reporting of measles cases to WHO, which is due, in part, to an underlying differential quality or sensitivity of case-based surveillance at country-level.^38^ Thus, the measles cases presented in the analysis might not reflect the true situation and makes intercountry comparison difficult. Fourth, we acknowledge that there might be some level of uncertainty around the estimated number of children who did not receive MCV1 in 2019. However, as there is no underlying probability model upon which the WUENIC estimates we used are based, it would be difficult to generate levels of uncertainty for the number of unvaccinated children. Finally, we did not include measles mortality data in this analysis and in the scorecard. Although reducing measles mortality is a key component of the measles elimination, our scorecard highlights readily available metrics that could be used to categorise, track, and compare country-level progress.

As it stands, measles will continue to be endemic in the west African region after 2020. In this already bleak landscape, the ongoing COVID-19 pandemic has resulted in disruptions of immunisation systems. There is, however, some hope as countries facing substantial challenges in reaching measles elimination milestones can learn what has worked from their neighbours like Cape Verde, The Gambia, and Ghana. This shared learning requires a coordinated regional effort, whereas countries must adapt such lessons to suit their local realities. Countries must prioritise the strengthening of their routine immunisation systems to ensure they rapidly increase the coverages of MCV1 and MCV2 that would provide an additional contact for other vaccination and child health services in the second year of life. Although this analysis showed that SIAs are impactful, they should be targeted at low coverage settings, migrants, and hard-to-reach subpopulations who are usually missed by the routine system.^26^ It is also imperative that countries strengthen their health systems and implement integrated strategies to deliver routine services and lifesaving interventions to address the health needs of these susceptible subpopulations. Furthermore, countries must strengthen measles case-based surveillance systems at subnational levels across the region to ensure rapid case detection and laboratory testing of suspected cases.^39^

## Data sharing

All data come from publicly available secondary data sources. Relevant country-level data supporting this study are uploaded as supplementary information. Following complete publication, additional data generated in this study will be made available to researchers on request to the corresponding author.

## References

[1] WHO (2019). More than 140,000 die from measles as cases surge worldwide. https://www.who.int/news/item/05-12-2019-more-than-140-000-die-from-measles-as-cases-surge-worldwide.

[2] Centers for Disease Control and Prevention (2020). Transmission of measles. https://www.cdc.gov/measles/transmission.html.

[3] Liu Y, Gayle AA, Wilder-Smith A, Rocklöv J (2020). The reproductive number of COVID-19 is higher compared to SARS coronavirus. J Travel Med.

[4] Khan A, Naveed M, Dur-E-Ahmad M, Imran M (2015). Estimating the basic reproductive ratio for the Ebola outbreak in Liberia and Sierra Leone. Infect Dis Poverty.

[5] Guerra FM, Bolotin S, Lim G (2017). The basic reproduction number (R_0_) of measles: a systematic review. Lancet Infect Dis.

[6] Mina MJ, Kula T, Leng Y (2019). Measles virus infection diminishes preexisting antibodies that offer protection from other pathogens. Science.

[7] WHO (2012). Global measles and rubella strategic plan 2012–2020.

[8] Masresha B, Luce R, Katsande R, Fall A, Eshetu M, Mihigo R (2017). The effect of targeted wide age range SIAs in reducing measles incidence in the African Region. Pan Afr Med J.

[9] Patel MK, Dumolard L, Nedelec Y (2019). Progress toward regional measles elimination worldwide, 2000–2018. MMWR Morb Mortal Wkly Rep.

[10] WHO Regional Office for Africa (2011). Measles elimination by 2020: a strategy for the African region report of the secretaria.

[11] UN News (2020). More international support needed to curb deadly measles outbreak in DR Congo. https://news.un.org/en/story/2020/01/1054941.

[12] Takahashi S, Metcalf CJ, Ferrari MJ (2015). Reduced vaccination and the risk of measles and other childhood infections post-Ebola. Science.

[13] Suk JE, Van Cangh T, Beauté J (2014). The interconnected and cross-border nature of risks posed by infectious diseases. Glob Health Action.

[14] Economic Community of West African States (2016). Member states. https://www.ecowas.int/member-states/.

[15] UN Department of Economic and Social Affairs Population Dynamics (2019). World population prospects 2019. https://population.un.org/wpp/Download/Standard/Population/.

[16] The World Bank World Bank country and lending groups. https://datahelpdesk.worldbank.org/knowledgebase/articles/906519-world-bank-country-and-lending-groups.

[17] Gavi, the Vaccine Alliance (2020). Africa. https://www.gavi.org/programmes-impact/country-hub/africa.

[18] WHO (2020). WHO-UNICEF estimates of MCV1 coverage. http://apps.who.int/immunization_monitoring/globalsummary/timeseries/tswucoveragemcv1.html.

[19] UNICEF Multiple Indicator Cluster Surveys (2021). Surveys. http://mics.unicef.org/surveys.

[20] The Demographic and Health Surveys Program Survey characteristics search. https://dhsprogram.com/what-we-do/survey-search.cfm?sendsearch=1&crt=1&listgrp=1.

[21] WHO (2020). Measles reported cases. http://apps.who.int/immunization_monitoring/globalsummary/timeseries/tsincidencemeasles.html.

[22] WHO Immunization, vaccines, and biologicals—data, statistics and graphs. https://www.who.int/immunization/monitoring_surveillance/data/en/.

[23] WHO (2005). Strengthening health security by implementing the International Health Regulations. https://www.who.int/ihr/publications/WHO_HSE_GCR_2018_2/en/.

[24] Arsenault C, Harper S, Nandi A, Rodríguez JM, Hansen PM, Johri M (2017). An equity dashboard to monitor vaccination coverage. Bull World Health Organ.

[25] WHO (2014). Immunization, vaccines and biologicals—WHO-recommended surveillance standard of measles. https://www.who.int/immunization/monitoring_surveillance/burden/vpd/surveillance_type/active/measles_standards/en/.

[26] Orenstein WA, Hinman A, Nkowane B, Olive JM, Reingold A (2018). Measles and rubella global strategic plan 2012-2020 midterm review. Vaccine.

[27] Wariri O, Edem B, Nkereuwem E (2019). Tracking coverage, dropout and multidimensional equity gaps in immunisation systems in West Africa, 2000–2017. BMJ Glob Health.

[28] Payne S, Townend J, Jasseh M, Lowe Jallow Y, Kampmann B (2014). Achieving comprehensive childhood immunization: an analysis of obstacles and opportunities in The Gambia. Health Policy Plan.

[29] WHO (2018). The state of health in the WHO African region: an analysis of the status of health, health services and health systems in the context of the Sustainable Development Goals.

[30] Government of Ghana (2014). Comprehensive multi-year plan for immunizations (2015–2019).

[31] UNICEF (2016). Two-thirds of unimmunized children live in conflict-affected countries. https://www.unicef.org/media/media_90987.html.

[32] Sato R (2019). Effect of armed conflict on vaccination: evidence from the Boko Haram insurgency in northeastern Nigeria. Confl Health.

[33] Truelove SA, Moss WJ, Lessler J (2015). Mitigating measles outbreaks in west Africa post-Ebola. Expert Rev Anti Infect Ther.

[34] Masresha BG, Luce R, Okeibunor J, Shibeshi ME, Kamadjeu R, Fall A (2018). Introduction of the second dose of measles containing vaccine in the childhood vaccination programs within the WHO Africa region—lessons learnt. J Immunol Sci.

[35] WHO Regional Office for Africa (2015). African regional guidelines for measles and rubella surveillance.

[36] Durrheim DN, Andrus JK, Pfaff G, Tabassum S, Bashour H, Githanga D (2019). Eradicating measles: a call for an exceptional coordinated global effort. J Infect Dis.

[37] National Primary Health Care Development Agency (2013). National Routine Immunization Strategic Plan 2013–2015.

[38] Fatiregun AA, Odega CC (2013). Representativeness of suspected measles cases reported in a southern district of Nigeria. Asian Pac J Trop Med.

[39] Masresha B, Katsande R, Luce R (2018). Performance of national measles case-based surveillance systems in the WHO African region. 2012–2016. J Immunol Sci.

