# Supplementary material for: A scorecard of progress towards measles elimination in 15 west African countries, 2001–19: a retrospective, multicountry analysis of national immunisation coverage and surveillance data
Source: Lancet Glob Health. 2021 Feb 16;9(3):e280–90. doi: 10.1016/S2214-109X(20)30481-2 (PMC7900524; doi:10.1016/S2214-109X(20)30481-2)
Supplement: Supplementary appendix [file mmc1.pdf]

THE LANCET
Global Health

Supplementary appendix

This appendix formed part of the original submission and has been peer reviewed. We post it as supplied by the authors.

Supplement to: Wariri O, Nkereuwem E, Erondu NA, et al. A scorecard of progress towards measles elimination in 15 west African countries, 2001–19: a retrospective, multicountry analysis of national immunisation coverage and surveillance data. *Lancet Glob Health* 2021; **9:** e280–90.

Supplementary appendix

A scorecard of progress towards measles elimination in 15 West African countries, 2001–2019: a retrospective, multicountry analysis of national immunisation coverage and surveillance data

Contents

Mini validation of scorecard 2

Benin Republic 3

Burkina Faso 4

Cote d'Ivoire 6

The Gambia 7

Ghana 8

Guinea 9

Guinea-Bissau 10

Liberia 11

Mali 12

Niger Republic 13

Nigeria 14

Senegal 15

Togo 17

Mini validation of scorecard

Supplementary Table 1: Validation of the country-level measles elimination scorecard based on data from countries in the WHO South East Asia region.

	MCV1 coverage		MCV2 coverage			Measles incidence rate/million				
**South-East Asian Country**	**Control target (2015)**	**Elimination target (2019)**	**Control target (2015)**	**Elimination target (2019)**	**Geographic equity gaps in MCV1 (in % points)**	**Control target (2015)**	**Elimination target (2019)**	**JEE scores**	**Country-level measles scorecard**	**WHO measles elimination status (Gold standard)**
**Bangladesh**	90	93	70	89	24.7	1.5	35.7	3	4	Not eliminated
**Bhutan**	97	97	94	92	***	15.0	2.6	5	6	**Eliminated***
**Indonesia**	87	88	31	71	***	58.4	6.7	4	3	Not eliminated
**Maldives**	99	100	100	100	15.4	0	0	5	8	**Eliminated***
**Myanmar**	84	84	78	80	31.9	0.1	97.0	3	4	Not eliminated
**Sri-Lanka**	99	99	99	99	6.9	75.0	2.3	5	7	**Eliminated***
**Thailand**	99	96	95	87	8	2.2	77.7	5	7	Not eliminated
**Timor-Leste**	95	85	0	80	37.2	36.0	17.0	4	4	**Eliminated***

*Countries that have been certified by the WHO to have officially eliminated measles

Note: *MCV1/2 coverage categories; (**green**= ≥90% in 2015 and ≥95% in 2018; **light green**= 80-89% in 2015 and 80-94% in 2018; **yellow**= 50-79%; **red**= ≤50%,). Countries yet to commence MCV2 as of July 2019 were highlighted red. Measles incidence rate/million categories; (**green**= <5 cases per million in 2015 and <1 case per million in 2018, **light green**= 5-9 cases per million in 2015 and 1-4 cases per million in 2018, **yellow**= 10-19 cases per million in 2015 and 5-9 cases per million in 2018, **red**= >20 cases per million in 2015 and >10 cases per million in 2018). *Intra-country geographic equity gap categories; (**green**= ≤20% points; **red**= >20% points). **JEE categories; (**red**=1, **yellow**=2-3, **green**=4-5). ***JEE yet to be conducted. Country-level measles scorecard categories; (**green**= achieved 6-8 out of the 8 measles summary indicators; **yellow**= achieved 3-5 out of the 8 measles summary indicators; and **red**= achieved only 1 or 2 out of the 8 measles summary indicators).*

Sensitivity of scorecard = 75%, **Specificity** of scorecard = 75%

Benin Republic

Supplementary Table 2: Total population, surviving infant population, MCV2 coverage and target population reached by SIAs in Benin Republic, 2001-2019.

	Years																		
**Country parameters**	**2001**	**2002**	**2003**	**2004**	**2005**	**2006**	**2007**	**2008**	**2009**	**2010**	**2011**	**2012**	**2013**	**2014**	**2015**	**2016**	**2017**	**2018**	**2019**
**Total population [x1000]**	7,079	7,295	7,520	7,750	7,982	8,216	8,452	8,696	8,944	9,199	9,460	9,729	10,004	10,286	10,575	10,872	11,175	11,485	11'801
**Surviving infant population [x1000]**	281	281	282	283	284	317	318	318	319	320	352	353	354	354	355	386	387	391	399
**MCV2 coverage (%) ***	0	0	0	0	0	0	0	0	0	0	0	0	0	0	0	0	0	0	0
**SIAs (% of target population reached]**	98		105		107.5			101			104			100	102				104

Note: *All SIAs (Supplementary Immunization Activities) conducted in Benin republic reached at least 95% of the targeted population between 2001-2019. Total population and surviving infant estimates are based on the United Nations (UN) World Population Prospects 2019. MCV2 and SIAs figures are based on WUENIC reported coverage. *Benin yet to commence MCV2 vaccination as of 2019.*

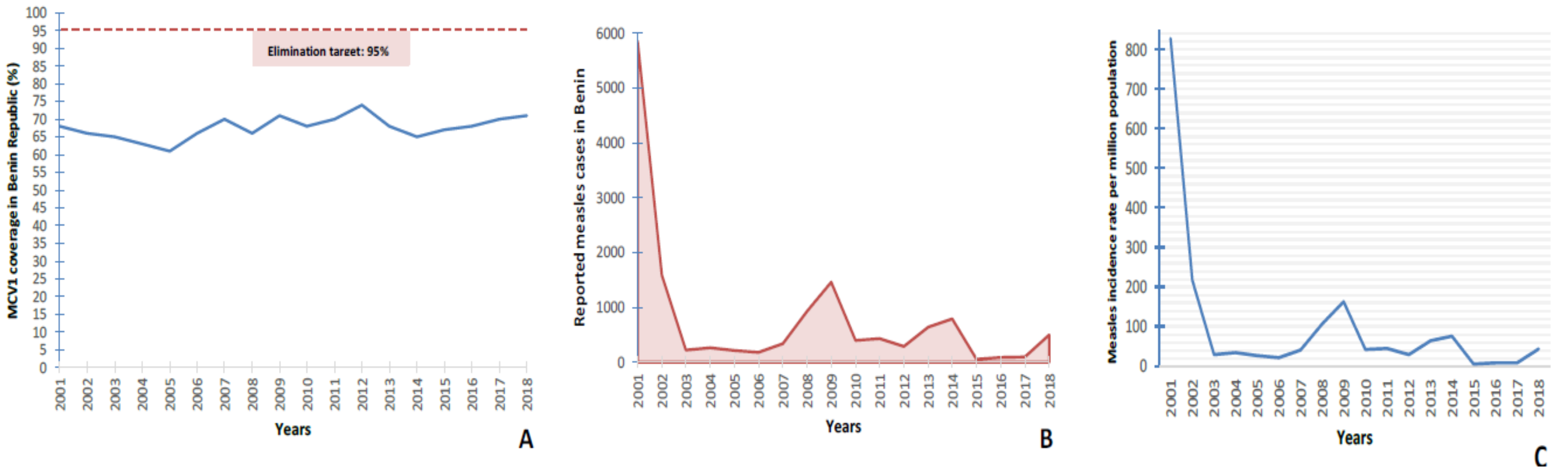

Supplementary figure 1: [A] MCV1 coverage rate in Benin, 2001-2018 [B] Reported measles cases in Benin, 2001-2018 [C] Measles incidence rate per million in Benin, 2001-2018

Burkina Faso

Supplementary Table 3: Total population, surviving infant population, MCV2 coverage and target population reached by SIAs in Burkina Faso, 2001-2019.

	Years																		
**Country parameters**	**2001**	**2002**	**2003**	**2004**	**2005**	**2006**	**2007**	**2008**	**2009**	**2010**	**2011**	**2012**	**2013**	**2014**	**2015**	**2016**	**2017**	**2018**	**2019**
**Total population [x1000]**	11,944	12,293	12,654	13,030	13,421	13,829	14,252	14,689	15,141	15,605	16,081	16,571	17,072	17,586	18,110	18,646	19,193	19,751	20'321
**Surviving infant population [x1000]**	519	520	521	523	524	585	587	588	590	592	644	645	647	661	673	686	698	711	723
**MCV2 coverage (%)**	0	0	0	0	0	0	0	0	0	0	0	0	0	17	50	59	65	71	71
**SIAs (% of target population reached]**	96			101			102		105		113	84		107				110	106

Note: *All SIAs (Supplementary Immunization Activities) conducted in Burkina Faso republic reached at least 95% of the targeted population between 2001-2019, except in 2012. Total population and surviving infant estimates are based on the United Nations (UN) World Population Prospects 2019. MCV2 and SIAs figures are based on WUENIC reported coverage.*

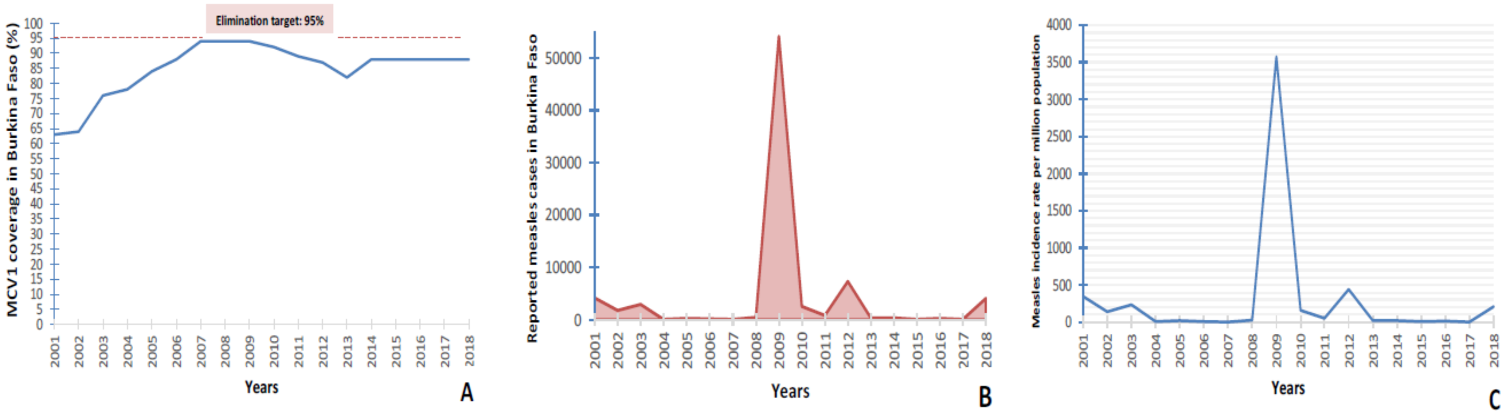

Supplementary figure 2: [A] MCV1 coverage rate in Burkina Faso, 2001-2018 [B] Reported measles cases in Burkina Faso, 2001-2018 [C] Measles incidence rate per million in Burkina Faso, 2001-2018.

Cape Verde

Supplementary Table 4: Total population, surviving infant population, MCV2 coverage and target population reached by SIAs in Cabo Verde, 2001-2019.

	Years																		
**Parameters**	**2001**	**2002**	**2003**	**2004**	**2005**	**2006**	**2007**	**2008**	**2009**	**2010**	**2011**	**2012**	**2013**	**2014**	**2015**	**2016**	**2017**	**2018**	**2019**
**Total population [x1000]**	436	443	449	456	463	469	475	480	486	492	498	505	511	518	524	531	537	543	550
**Surviving infant population [x1000]**	12	12	12	12	12	11	11	11	11	11	11	11	11	11	11	11	11	10	10
**MCV2 coverage**	0	0	0	0	0	0	0	0	0	0	99	94	89	79	95	92	85	88	91
**SIAs (% of target population reached)**					93				87				95					96	

Note: *SIAs (Supplementary Immunization Activities) conducted in Cabo Verde reached at least 95% of the targeted population in 2013 and 2019, except in 2005 and 2009. Total population and surviving infant estimates are based on the United Nations (UN) World Population Prospects 2019. MCV2 and SIAs figures are based on WUENIC reported coverage.*

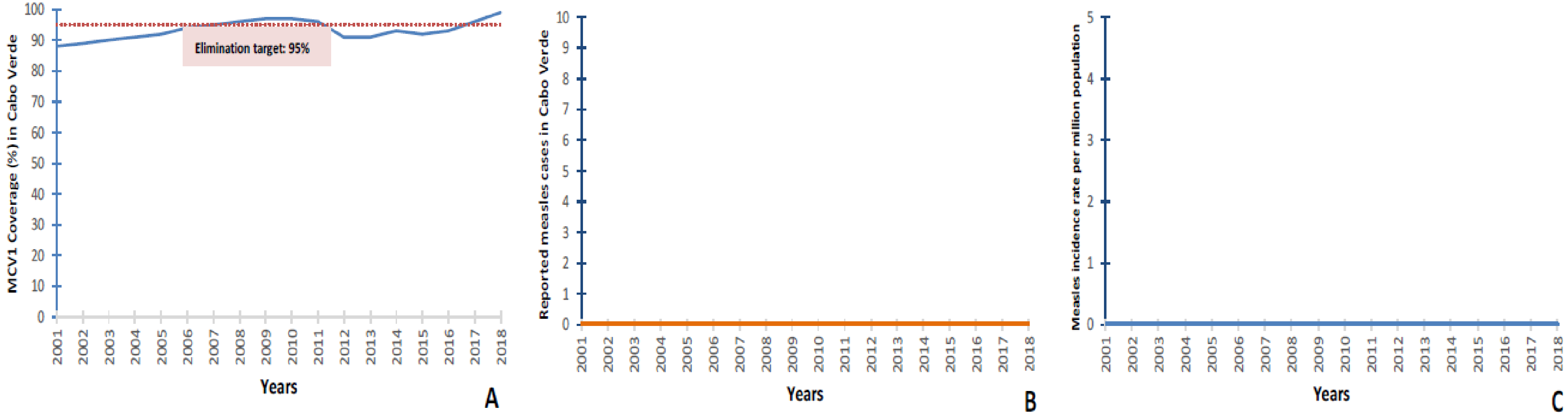

Supplementary figure 3: [A] MCV1 coverage rate in Cape Verde, 2001-2018 [B] Reported measles cases in Cape Verde, 2001-2018 [C] Measles incidence rate per million in Cape Verde, 2001-2018.

Cote d'Ivoire

Supplementary Table 5: Total population, surviving infant population, MCV2 coverage and target population reached by SIAs in Cote d'Ivoire, 2001-2019.

	Years																		
**Parameters**	**2001**	**2002**	**2003**	**2004**	**2005**	**2006**	**2007**	**2008**	**2009**	**2010**	**2011**	**2012**	**2013**	**2014**	**2015**	**2016**	**2017**	**2018**	**2019**
**Total population [x1000]**	16,853	17,231	17,599	17,970	18,354	18,754	19,171	19,605	20,059	20,532	21,028	21,547	22,087	22,647	23,226	23,822	24,437	25,069	25'717
**Surviving infant population [x1000]**	633	635	636	638	640	685	687	689	690	692	758	760	761	778	794	811	828	844	860
**MCV2 coverage**	0	0	0	0	0	0	0	0	0	0	0	0	0	0	0	0	0	0	0
**SIAs (% of target population reached)**			89		85			95			95			92				98	

Note: *SIAs (Supplementary Immunization Activities) conducted in Cote d'Ivoire reached at least 95% of the targeted population in 2008, 2011 and 2019, except in 2003, 2005 and 2014. Total population and surviving infant estimates are based on the United Nations (UN) World Population Prospects 2019. MCV2 and SIAs figures are based on WUENIC reported coverage.*

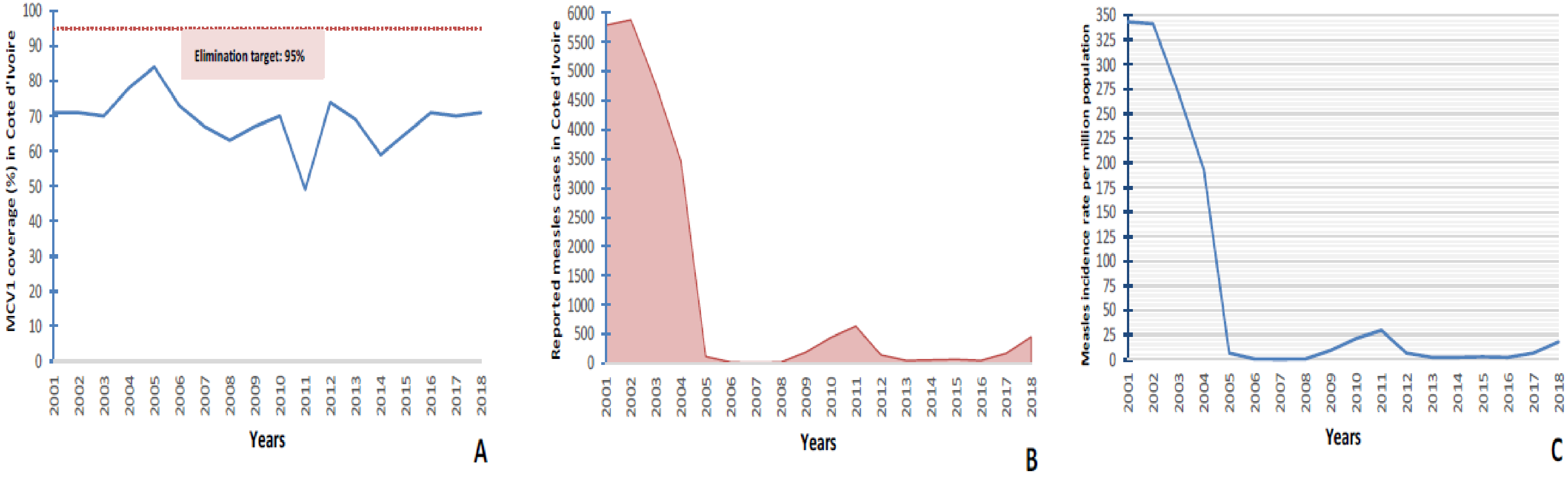

Supplementary figure 4: [A] MCV1 coverage rate in Cote d'Ivoire, 2001-2018 [B] Reported measles cases in Cote d'Ivoire, 2001-2018 [C] Measles incidence rate per million in Cote d'Ivoire, 2001-2018.

The Gambia

Supplementary Table 6: Total population, surviving infant population, MCV2 coverage and target population reached by SIAs in The Gambia, 2001-2019.

	Years																		
**Parameters**	**2001**	**2002**	**2003**	**2004**	**2005**	**2006**	**2007**	**2008**	**2009**	**2010**	**2011**	**2012**	**2013**	**2014**	**2015**	**2016**	**2017**	**2018**	**2019**
**Total population [x1000]**	1,360	1,404	1,449	1,496	1,543	1,591	1,639	1,689	1,740	1,793	1,848	1,905	1,963	2,024	2,085	2,149	2,213	2,280	2'348
**Surviving infant population [x1000]**	56	56	56	56	56	61	63	63	64	64	72	72	73	77	79	81	82	84	86
**MCV2 coverage**	0	0	0	0	0	0	0	0	0	0	0	56	53	73	81	79	68	71	61
**SIAs (% of target population reached)**							96				95					97			

Note: *All SIAs (Supplementary Immunization Activities) conducted in The Gambia reached at least 95% of the targeted population in 2007, 2011 and 2016. Total population and surviving infant estimates are based on the United Nations (UN) World Population Prospects 2019. MCV2 and SIAs estimates are based on WUENIC reported coverage.*

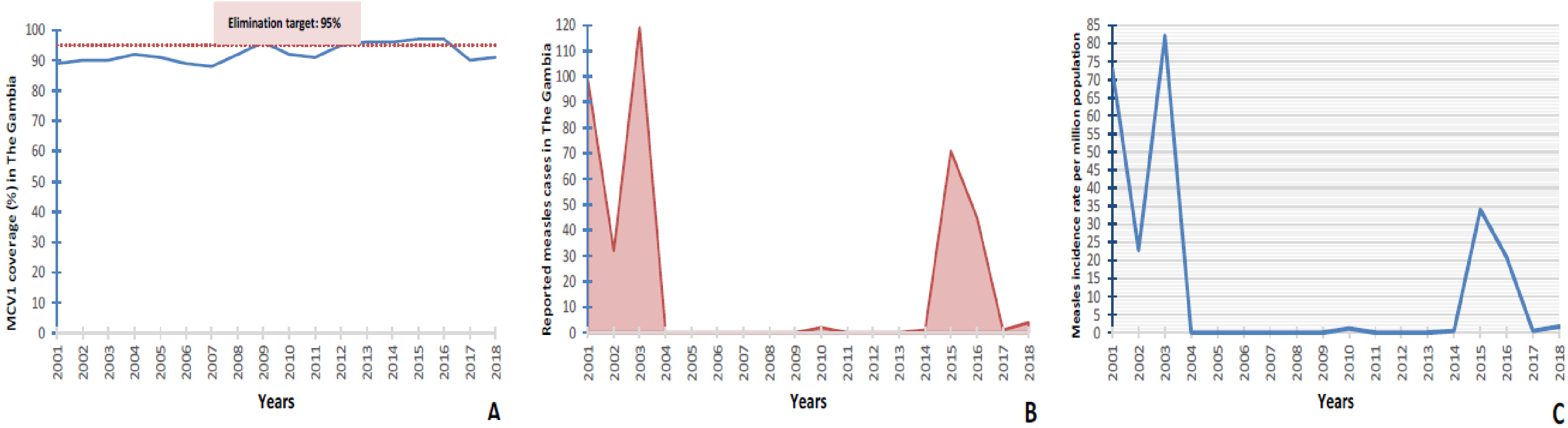

Supplementary figure 5: [A] MCV1 coverage rate in The Gambia, 2001-2018 [B] Reported measles cases in The Gambia, 2001-2018 [C] Measles incidence rate per million in The Gambia, 2001-2018.

Ghana

Supplementary Table 7: Total population, surviving infant population, MCV2 coverage and target population reached by SIAs in Ghana, 2001-2019.

	Years																		
**Parameters**	**2001**	**2002**	**2003**	**2004**	**2005**	**2006**	**2007**	**2008**	**2009**	**2010**	**2011**	**2012**	**2013**	**2014**	**2015**	**2016**	**2017**	**2018**	**2019**
**Total population [x1000]**	19,756	20,246	20,750	21,272	21,814	22,379	22,963	23,563	24,170	24,779	25,387	25,996	26,607	27,224	27,849	28,481	29,121	29,767	30'418
**Surviving infant population [x1000]**	657	658	659	660	661	733	734	735	736	738	812	814	815	812	822	830	838	8 45	853
**MCV2 coverage**	0	0	0	0	0	0	0	0	0	0	0	52	54	67	63	75	83	83	83
**SIAs (% of target population reached)**	99	102				79				93			99					98	

Note: *SIAs (Supplementary Immunization Activities) conducted in Ghana reached at least 95% of the targeted population in 2001, 2002, 2013 and 2019, except in 2006 and 2010. Total population and surviving infant estimates are based on the United Nations (UN) World Population Prospects 2019. MCV2 and SIAs figures are based on WUENIC reported coverage.*

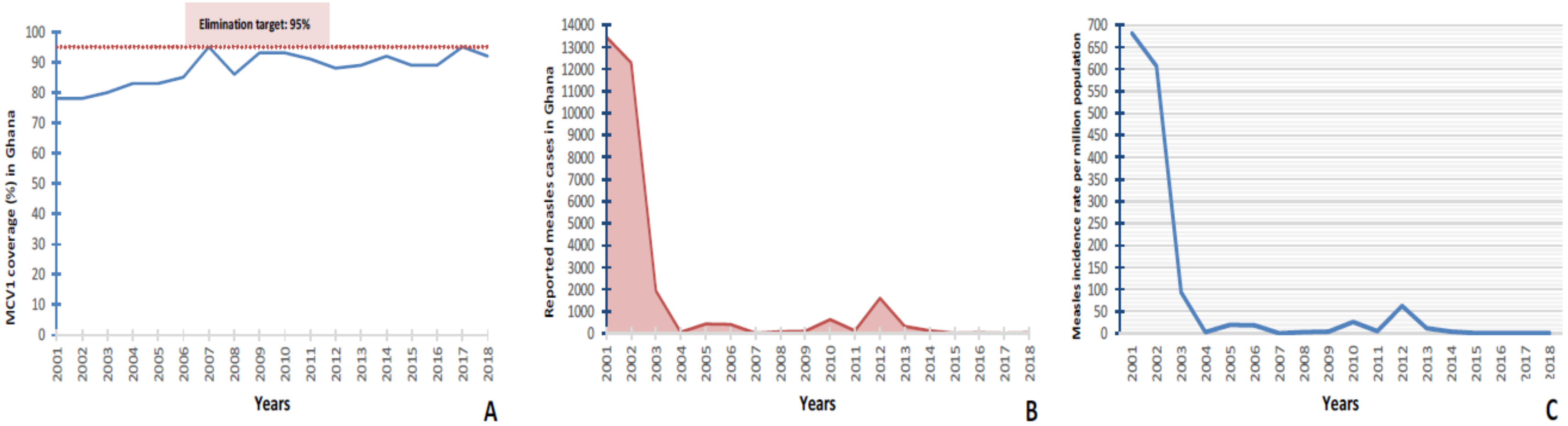

Supplementary figure 6: [A] MCV1 coverage rate in The Ghana, 2001-2018 [B] Reported measles cases in The Ghana, 2001-2018 [C] Measles incidence rate per million in The Ghana, 2001-2018.

Guinea

Supplementary Table 8: Total population, surviving infant population, MCV2 coverage and target population reached by SIAs in Guinea, 2001-2019.

	Years																		
**Parameters**	**2001**	**2002**	**2003**	**2004**	**2005**	**2006**	**2007**	**2008**	**2009**	**2010**	**2011**	**2012**	**2013**	**2014**	**2015**	**2016**	**2017**	**2018**	**2019**
**Total population [x1000]**	8,417	8,586	8,753	8,925	9,109	9,307	9,518	9,738	9,964	10,192	10,420	10,652	10,892	11,150	11,432	11,738	12,067	12,414	12'771
**Surviving infant population [x1000]**	3489	350	352	353	355	374	375	376	377	378	384	394	399	400	407	414	422	330	437
**MCV2 coverage**	0	0	0	0	0	0	0	0	0	0	0	0	0	0	0	0	0	0	0
**SIAs (% of target population reached)**		98	98			97			101			95		99	91	103	104*		

Note: *SIAs (Supplementary Immunization Activities) conducted in Guinea reached at least 95% of the targeted population in 2002, 2003, 2006, 2009, 2012, 2014, 2016 and 2017, except in 2015. Total population and surviving infant figures are based on the United Nations (UN) World Population Prospects 2019. MCV2 and SIAs estimates are based on WUENIC reported coverage. *Three SIAs were conducted in 2017 and all reached more than 95% of targeted population.*

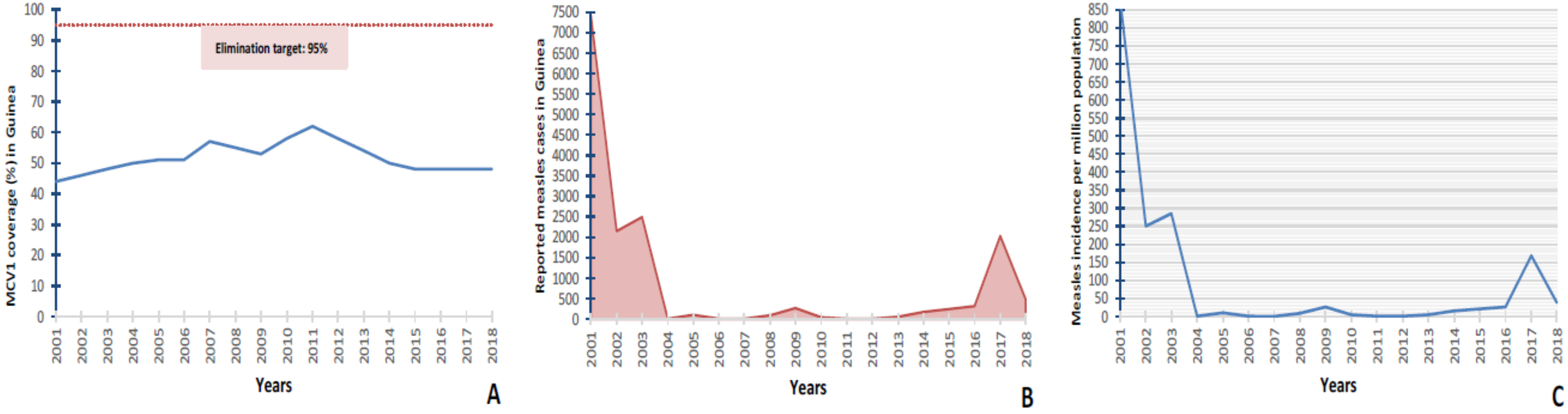

Supplementary figure 7: [A] MCV1 coverage rate in The Guinea, 2001-2018 [B] Reported measles cases in The Guinea, 2001-2018 [C] Measles incidence rate per million in The Guinea, 2001-2018.

Guinea-Bissau

Supplementary Table 9: Total population, surviving infant population, MCV2 coverage and target population reached by SIAs in Guinea-Bissau, 2001-2019.

	Years																		
**Parameters**	**2001**	**2002**	**2003**	**2004**	**2005**	**2006**	**2007**	**2008**	**2009**	**2010**	**2011**	**2012**	**2013**	**2014**	**2015**	**2016**	**2017**	**2018**	**2019**
**Total population [x1000]**	1,227	1,254	1,283	1,313	1,344	1,377	1,411	1,446	1,483	1,522	1,562	1,604	1,648	1,692	1,737	1,782	1,828	1,874	1'921
**Surviving infant population [x1000]**	47	48	48	48	47	53	53	54	54	56	57	58	59	59	60	61	62	62	63
**MCV2 coverage**	0	0	0	0	0	0	0	0	0	0	0	0	0	0	0	0	0	0	0
**SIAs (% of target population reached)**						91			101			89			86				83

Note: *SIAs (Supplementary Immunization Activities) conducted in Guinea-Bissau only reached at least 95% of the targeted population in 2009. Total population and surviving infant figures are based on the United Nations (UN) World Population Prospects 2019. MCV2 and SIAs estimates are based on WUENIC reported coverage.*

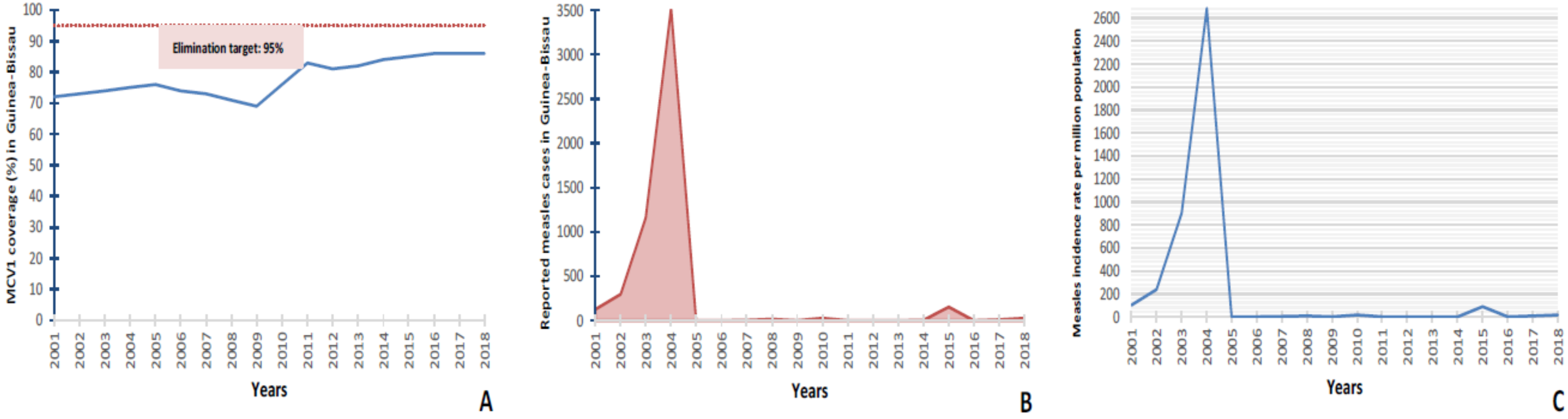

Supplementary figure 8: [A] MCV1 coverage rate in Guinea-Bissau, 2001-2018 [B] Reported measles cases in Guinea-Bissau, 2001-2018 [C] Measles incidence rate per million in Guinea-Bissau, 2001-2018.

Liberia

Supplementary Table 10: Total population, surviving infant population, MCV2 coverage and target population reached by SIAs in Liberia, 2001-2019.

	Years																		
**Parameters**	**2001**	**2002**	**2003**	**2004**	**2005**	**2006**	**2007**	**2008**	**2009**	**2010**	**2011**	**2012**	**2013**	**2014**	**2015**	**2016**	**2017**	**2018**	**2019**
**Total population [x1000]**	2,954	3,024	3,077	3,135	3,218	3,329	3,462	3,608	3,754	3,891	4,017	4,135	4,248	4,359	4,472	4,586	4,702	4,818	4'937
**Surviving infant population [x1000]**	113	113	114	115	116	117	117	118	118	119	141	141	142	143	145	147	149	151	153
**MCV2 coverage**	0	0	0	0	0	0	0	0	0	0	0	0	0	0	0	0	0	0	13
**SIAs (% of target population reached)**				105			97	95		103	103				99			97	

Note: *All SIAs (Supplementary Immunization Activities) conducted in Liberia reached at least 95% of the targeted population. Total population and surviving infant estimates are based on the United Nations (UN) World Population Prospects 2019. MCV2 and SIAs estimates are based on WUENIC reported coverage.*

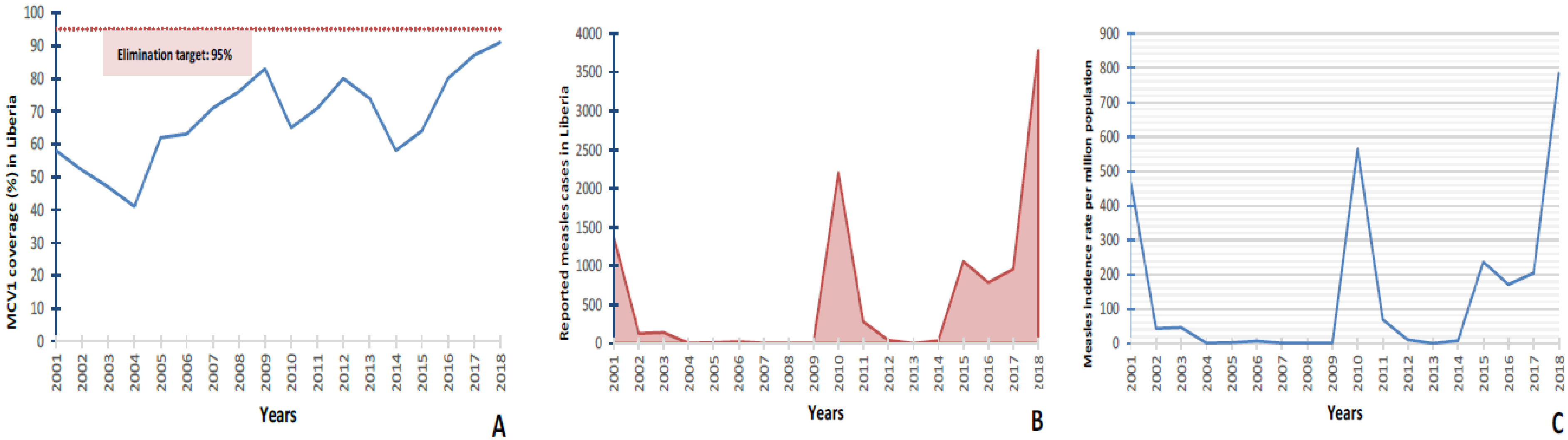

Supplementary figure 9: [A] MCV1 coverage rate in Liberia, 2001-2018 [B] Reported measles cases in Liberia, 2001-2018 [C] Measles incidence rate per million in Liberia, 2001-2018.

Mali

Supplementary Table 11: Total population, surviving infant population, MCV2 coverage and target population reached by SIAs in Mali, 2001-2019.

	Years																		
**Parameters**	**2001**	**2002**	**2003**	**2004**	**2005**	**2006**	**2007**	**2008**	**2009**	**2010**	**2011**	**2012**	**2013**	**2014**	**2015**	**2016**	**2017**	**2018**	**2019**
**Total population [x1000]**	11,271	11,617	11,983	12,369	12,775	13,203	13,651	14,113	14,581	15,049	15,514	15,979	16,449	16,934	17,439	17,965	18,512	19,078	19'658
**Surviving infant population [x1000]**	514	516	519	521	524	604	606	608	610	612	672	674	675	689	702	715	729	743	758
**MCV2 coverage**	0	0	0	0	0	0	0	0	0	0	0	0	0	0	0	0	0	0	4
**SIAs (% of target population reached)**	99			118			101				92	95*			112				109

Note: *SIAs (Supplementary Immunization Activities) conducted in Mali reached at least 95% of the targeted population in 2001, 2004, 2007, 2012, and 2015, except in 2011. Total population and surviving infant figures are based on the United Nations (UN) World Population Prospects 2019. MCV2 and SIAs estimates are based on WUENIC reported coverage. *Two SIAs were conducted in 2012 and both reached more least 95% of targeted population.*

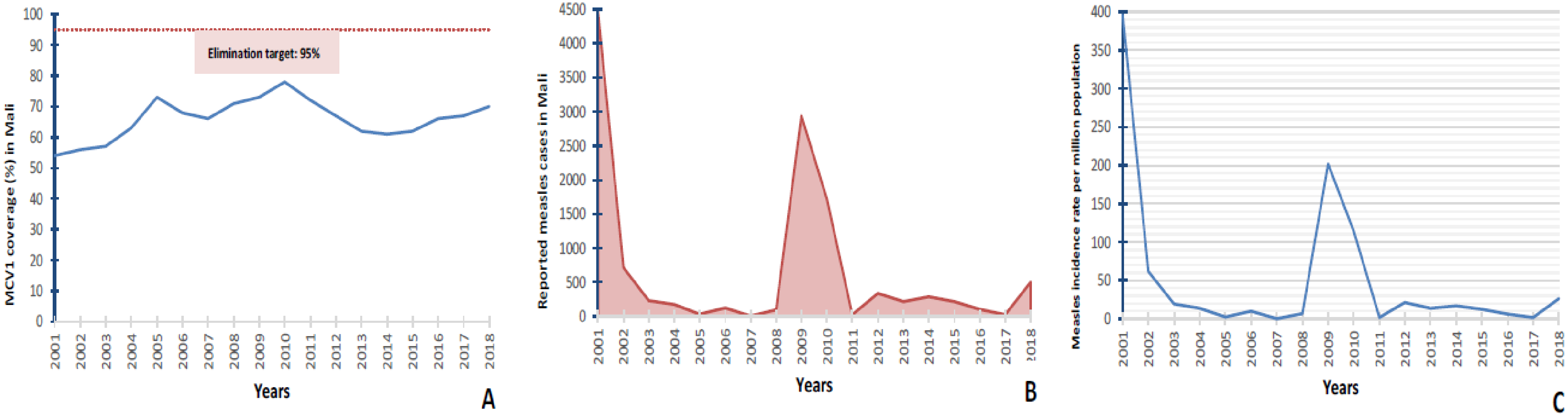

Supplementary figure 10: [A] MCV1 coverage rate in Mali, 2001-2018 [B] Reported measles cases in Mali, 2001-2018 [C] Measles incidence rate per million in Mali, 2001-2018.

Niger Republic

Supplementary Table 12: Total population, surviving infant population, MCV2 coverage and target population reached by SIAs in Niger Republic, 2001-2019.

	Years																		
**Parameters**	**2001**	**2002**	**2003**	**2004**	**2005**	**2006**	**2007**	**2008**	**2009**	**2010**	**2011**	**2012**	**2013**	**2014**	**2015**	**2016**	**2017**	**2018**	**2019**
**Total population [x1000]**	11,751	12,189	12,647	13,125	13,624	14,143	14,685	15,250	15,843	16,464	17,114	17,795	18,504	19,240	20,001	20,788	21,602	22,442	23'311
**Surviving infant population [x1000]**	596	599	601	604	607	708	709	712	714	717	840	842	844	878	905	932	961	990	1'020
**MCV2 coverage**	0	0	0	0	0	0	0	0	0	0	0	0	0	3	16	31	46	48	58
**SIAs (% of target population reached)**				99	102			100		102		102			96				102

Note: *All SIAs (Supplementary Immunization Activities) conducted in Niger Republic reached at least 95% of the targeted population. Total population and surviving infant estimates are based on the United Nations (UN) World Population Prospects 2019. MCV2 and SIAs estimates are based on WUENIC reported coverage.*

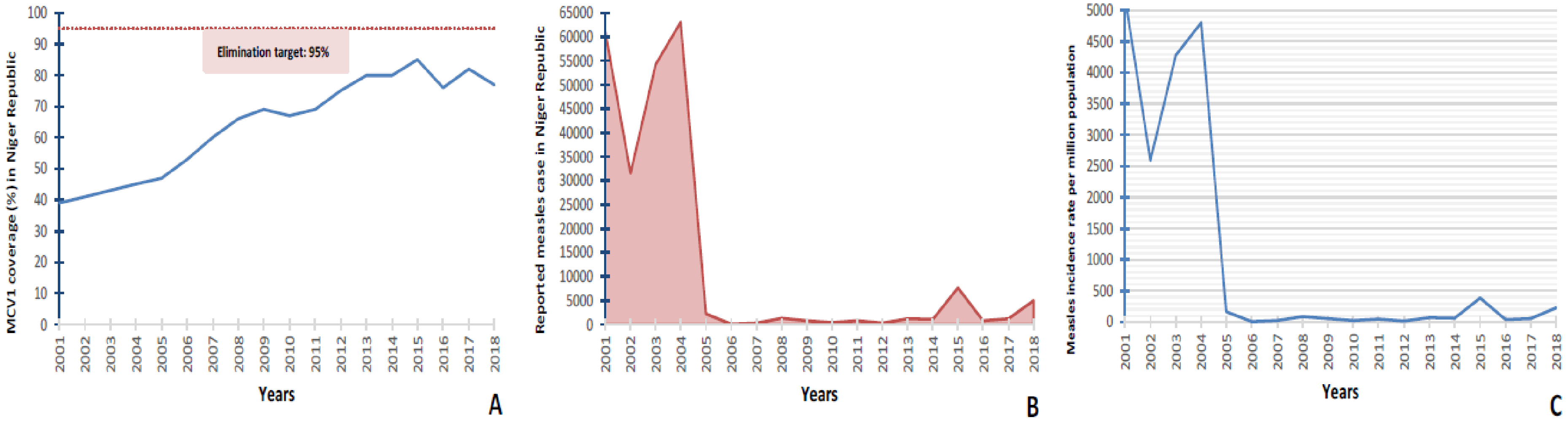

Supplementary figure 11: [A] MCV1 coverage rate in Niger Republic, 2001-2018 [B] Reported measles cases in Niger Republic, 2001-2018 [C] Measles incidence rate per million in Niger Republic, 2001-2018.

Nigeria

Supplementary Table 13: Total population, surviving infant population, MCV2 coverage and target population reached by SIAs in Nigeria, 2001-2019.

	Years																		
Parameters	**2001**	**2002**	**2003**	**2004**	**2005**	**2006**	**2007**	**2008**	**2009**	**2010**	**2011**	**2012**	**2013**	**2014**	**2015**	**2016**	**2017**	**2018**	**2019**
**Total population [x1000]**	125,394	128,596	131,900	135,320	138,865	142,538	146,339	150,269	154,324	158,503	162,805	167,228	171,765	176,404	181,137	185,960	190,873	195,874	200'964
**Surviving infant population [x1000]**	5,002	5,019	5,036	5,053	5,071	5,659	5,678	5,697	57,159	57,340	6,345	6,363	63,801	6,537	6,651	6,761	6,869	6,977	7'086
**MCV2 coverage**	0	0	0	0	0	0	0	0	0	0	0	0	0	0	0	0	0	0	9
**SIAs (% of target population reached)**					95	83	89*	97			100		107*		100	131	107*	106	104

Note: *SIAs (Supplementary Immunization Activities) conducted in Nigeria reached at least 95% of the targeted population in 2005, 2008, 2011, 2013, 2015, 2016, 2017 and 2018, except in 2007. Total population and surviving infant figures are based on the United Nations (UN) World Population Prospects 2019. MCV2 and SIAs estimates are based on WUENIC reported coverage. *Two SIAs each were conducted in 2007 and 2017, while three were conducted in 2013.*

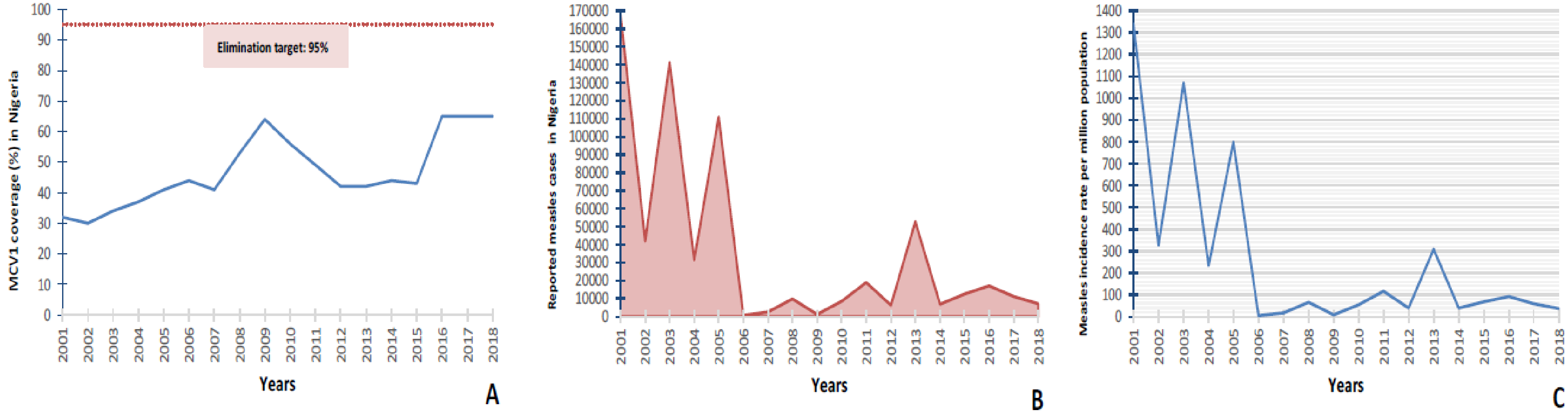

Supplementary figure 12: [A] MCV1 coverage rate in Nigeria, 2001-2018 [B] Reported measles cases in Nigeria, 2001-2018 [C] Measles incidence rate per million in Nigeria, 2001-2018.

Senegal

Supplementary Table 14: Total population, surviving infant population, MCV2 coverage and target population reached by SIAs in Senegal, 2001-2019.

	Years																		
**Parameters**	**2001**	**2002**	**2003**	**2004**	**2005**	**2006**	**2007**	**2008**	**2009**	**2010**	**2011**	**2012**	**2013**	**2014**	**2015**	**2016**	**2017**	**2018**	**2019**
**Total population [x1000]**	10,036	10,283	10,541	10,810	11,090	11,382	11,687	12,004	12,335	12,678	13,033	13,401	13,782	14,174	14,578	14,993	15,419	15,854	16'296
**Surviving infant population [x1000]**	382	383	385	386	3,874	441	442	443	444	445	502	503	504	504	511	518	524	530	537
**MCV2 coverage**	0	0	0	0	0	0	0	0	0	0	0	0	0	13	54	75	70	63	78
**SIAs (% of target population reached)**			98			99				81			101				107		

Note: *All SIAs (Supplementary Immunization Activities) conducted in Senegal reached at least 95% of the targeted population, except in 2010. Total population and surviving infant estimates are based on the United Nations (UN) World Population Prospects 2019. MCV2 and SIAs estimates are based on WUENIC reported coverage.*

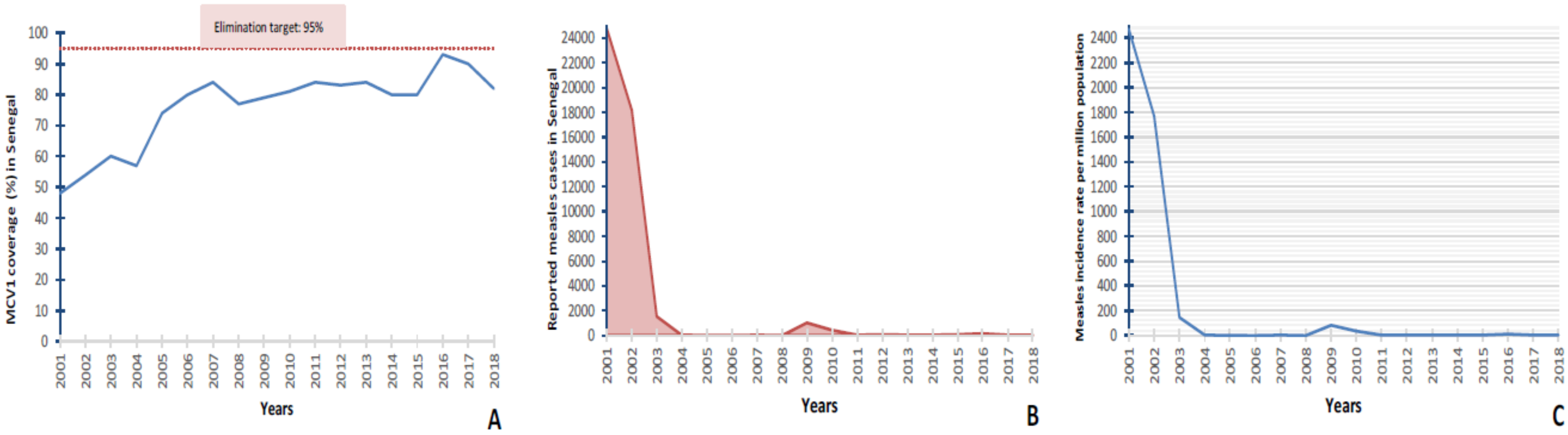

Supplementary figure 13: [A] MCV1 coverage rate in Senegal, 2001-2018 [B] Reported measles cases in Senegal, 2001-2018 [C] Measles incidence rate per million in Senegal, 2001-2018.

Sierra Leone

Supplementary Table 15: Total population, surviving infant population, MCV2 coverage and target population reached by SIAs in Sierra Leone, 2001-2019.

	Years																		
**Parameters**	**2001**	**2002**	**2003**	**2004**	**2005**	**2006**	**2007**	**2008**	**2009**	**2010**	**2011**	**2012**	**2013**	**2014**	**2015**	**2016**	**2017**	**2018**	**2019**
**Total population [x1000]**	4,754	4,965	5,201	5,433	5,645	5,829	5,989	6,133	6,272	6,415	6,563	6,712	6,863	7,017	7,172	7,329	7,488	7,650	7'813
**Surviving infant population [x1000]**	196	196	197	198	199	221	222	223	224	225	227	228	228	228	230	232	234	236	237
**MCV2 coverage**	0	0	0	0	0	0	0	0	0	0	0	0	0	0	60	50	55	64	72
**SIAs (% of target population reached)**			93			100			101			102			97*	100		105	99

Note: *All SIAs (Supplementary Immunization Activities) conducted in Senegal reached at least 95% of the targeted population, except in 2003. Total population and surviving infant estimates are based on the United Nations (UN) World Population Prospects 2019. MCV2 and SIAs estimates are based on WUENIC reported coverage. *Two SIAs were conducted in 2015 and both reached at least 95% of the target population.*

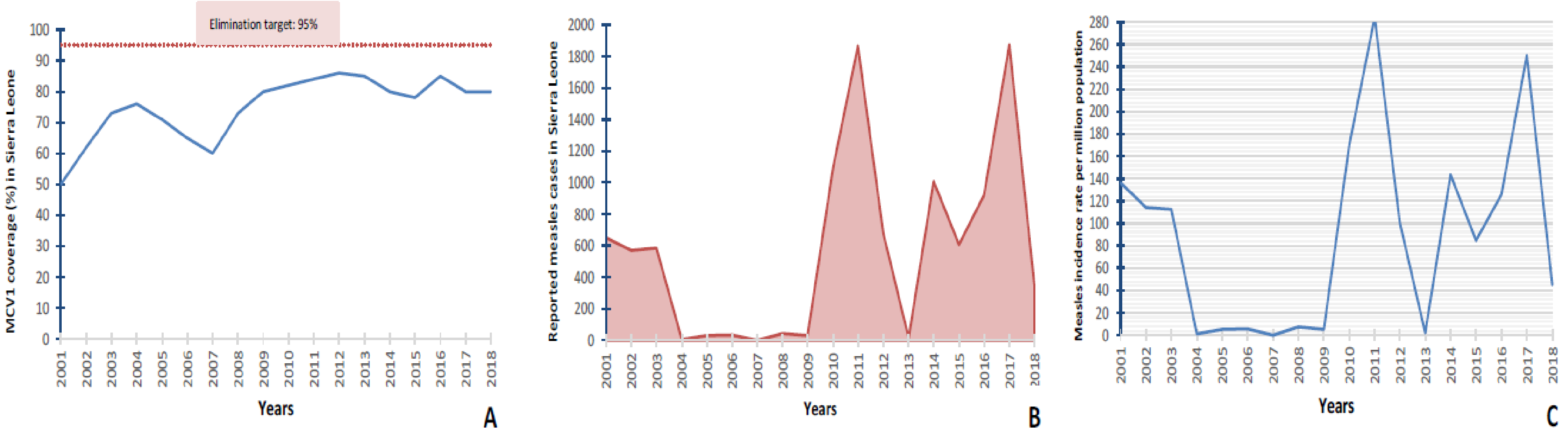

Supplementary figure 14: [A] MCV1 coverage rate in Sierra Leone, 2001-2018 [B] Reported measles cases in Sierra Leone, 2001-2018 [C] Measles incidence rate per million in Sierra Leone, 2001-2018.

Togo

Supplementary Table 16: Total population, surviving infant population, MCV2 coverage and target population reached by SIAs in Togo, 2001-2019.

	Years																		
**Parameters**	**2001**	**2002**	**2003**	**2004**	**2005**	**2006**	**2007**	**2008**	**2009**	**2010**	**2011**	**2012**	**2013**	**2014**	**2015**	**2016**	**2017**	**2018**	**2019**
**Total population [x1000]**	5,062	5,197	5,330	5,467	5,611	5,762	5,920	6,083	6,250	6,421	6,595	6,773	6,954	7,137	7,323	7,509	7,698	7,889	8'082
**Surviving infant population [x1000]**	195	195	195	196	197	218	218	219	219	219	235	235	235	237	240	242	246	249	252
**MCV2 coverage**	0	0	0	0	0	0	0	0	0	0	0	0	0	0	0	0	0	0	67
**SIAs (% of target population reached)**	99			100				98		97			96		99			98	

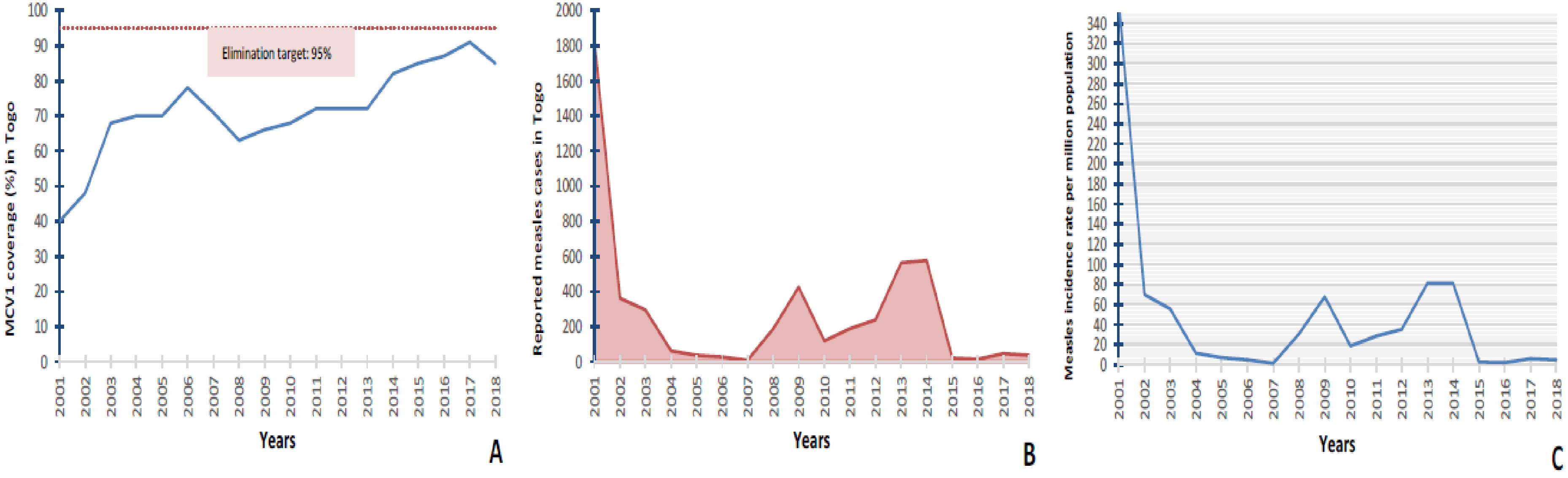

Supplementary figure 15: [A] MCV1 coverage rate in Togo, 2001-2018 [B] Reported measles cases in Togo, 2001-2018 [C] Measles incidence rate per million in Togo, 2001-2018.
